# Muscle Weakness and the Irisin–BDNF and Oxidative Stress Axis in the 60‐Day Pseudorandomised Controlled AGBRESA Bed Rest Study

**DOI:** 10.1002/jcsm.70250

**Published:** 2026-03-24

**Authors:** Alessandra Bosutti, Bergita Ganse, Edwin Mulder, Markus Gruber, Maria Venegas‐Carro, Jochen Zange, Jörn Rittweger, Moritz Eggelbusch, Rob C. I. Wüst, Paul Hendrickse, Hans Degens

**Affiliations:** ^1^ Department of Life Sciences University of Trieste Trieste Italy; ^2^ Innovative Implant Development (Fracture Healing), Departments and Institutes of Surgery Saarland University Homburg Germany; ^3^ German Aerospace Centre (DLR) Institute of Aerospace Medicine Cologne Germany; ^4^ Human Performance Research Centre, Department of Sport Science University Konstanz Konstanz Germany; ^5^ Applied Medical Informatics Tübingen University Hospital Tübingen Germany; ^6^ Department of Human Movement Sciences, Faculty of Behavioural and Movement Sciences Vrije Universiteit Amsterdam Amsterdam The Netherlands; ^7^ Professorship of Exercise Biology, Department Health and Sport Sciences, TUM School of Medicine and Health Technical University of Munich Munich Germany; ^8^ Lancaster Medical School Lancaster University Lancaster UK; ^9^ Department of Life Sciences Manchester Metropolitan University Manchester UK; ^10^ Institute of Sport Science and Innovations Lithuanian Sports University Kaunas Lithuania

**Keywords:** artificial gravity, irisin, microgravity, muscle atrophy, muscle contractile properties, muscle strength, oxidative stress, spaceflight

## Abstract

**Background:**

Muscle atrophy and weakness are among the most detrimental consequences of disuse, microgravity, hospitalisation and ageing. Oxidative modifications of myofibrillar proteins generated by oxidative stress may contribute to the reduced force‐ and power‐generating capacity of skeletal muscles. As part of the 60‐day AGBRESA bed rest (BR) study, we studied (1) how microgravity‐induced disuse affected markers of systemic and muscle oxidative stress, (2) how these related to muscle function and (3) to what extent artificial gravity (AG) attenuated these changes. Since the myokine irisin may protect against muscle deterioration in disuse, we additionally assessed serum irisin levels.

**Methods:**

Sixteen men and eight women (33 ± 9 years) participated in the AGBRESA study. Participants were pseudorandomly assigned to a control group (BR only), or a continuous or intermittent centrifugation group (*n* = 8 in each group) to assess the efficacy of daily 30‐min AG in attenuating the adverse effects of BR‐induced disuse. Muscle function, muscle protein carbonyls, serum irisin and key modulators of oxidative stress and cell protection in muscle and blood were assessed before, on Day 6, and at the end of BR.

**Results:**

BR caused a reduction in peak torque during maximal voluntary isometric knee extension and knee flexion (*p* **<** 0.001) that was greater in women than in men (knee extension, w: −39.7 ± 3.5%, m: −25.1 ± 2.4%; knee flexion, w: −32.9 ± 4.5%, m: −10.2 ± 3.5%, *p ≤* 0.002) and faster electrically evoked twitch muscle contractions of plantar flexor and knee extensor muscles (half relaxation time and % peak rate of relaxation, *p* ≤ 0.003). AG attenuated the BR‐induced increase in evoked twitch contraction speed in the knee extensors (group × time interactions: half relaxation time, *p* = 0.009; % peak rate of relaxation, *p* = 0.030), and the loss of evoked twitch peak torque of plantar flexors (AG − 25%, Controls −48%, group × time interactions, *p* = 0.020). Neither BR nor AG affected the circulating levels of systemic oxidative stress and muscle carbonyl concentration and serum irisin levels. However, participants with the highest serum irisin and brain‐derived neurotrophic factor levels showed lower levels of 8‐iso‐PGF2α, a marker of systemic oxidative stress (*r* = −0.486, *p* = 0.019; *r* = −0.512, *p* = 0.012, respectively) and circulating levels of the C‐terminal agrin fragment, a biomarker of neuromuscular junction fragmentation.

**Conclusions:**

AG exposure attenuated some of the BR‐induced changes in twitch contractile properties. Neither BR nor AG induced significant alterations in systemic oxidative stress, or muscle protein carbonylation, suggesting that the main contribution to the BR‐induced loss of muscle strength during the AGBRESA study was not oxidative stress.

## 1 Introduction

Skeletal muscle atrophy still poses major health risks during bed rest (BR), in hospital settings and human space missions. In microgravity, unloading causes a loss of muscle mass, a slow‐to‐fast fibre type transition and a decreased capacity of aerobic energy metabolism that, over time, causes a reduction in muscle strength and an earlier onset of muscle fatigue [1, 2, 3, S1]. The reduction in oxidative capacity contributes largely to a decreased muscle fatigue resistance [1, 2], while a reduction in muscle strength in response to disuse is mostly due to muscle atrophy, and is detectable already in the early stages of disuse. The loss of muscle strength exceeds the loss of muscle mass, suggesting that additional factors, such as a reduction in the voluntary muscle activation capacity [S2, S3], contribute to the development of muscle weakness [1, 2, 3, 4, S1‐S3]. Another potential factor is oxidative stress, which may promote muscle weakness via oxidative modifications of myofibrillar proteins [5, 6, S4]. Oxidative stress could also contribute to muscle weakness by inducing a decline in neuromuscular junction (NMJ) integrity and impairing excitation–contraction coupling. In disuse, reductions in NMJ integrity impair postsynaptic signal transduction that may, over time, result in a reduction in the number of motor units and an increase in motor unit size that in turn result in an impaired force‐generating capacity [3, S2, S3].

Although the impact of BR on the oxidant and antioxidant status has been investigated in several studies, its implications remain equivocal, as studies have seen increased [7, 8, 9], unchanged [8, 10, S5] or even decreased [S6] levels of oxidative stress and/or oxidative modifications of muscle proteins. Part of the discrepancies between studies may be related to methodological differences, for example in the timing of the measurement after the start of BR. In fact, we suggest that the divergent results may hint to temporal variation of oxidative stress in response to disuse. So far, the time course of the systemic and muscle‐specific oxidative stress response to BR in the same individuals has yet to be clarified. Furthermore, it remains to be seen, whether there is a relationship between muscle function and systemic and muscle oxidative stress after BR. If so, the modulation of oxidative stress would be a potential target for countermeasures to attenuate BR‐induced muscle weakness.

Muscle disuse has also been reported to diminish the release of neurotrophic factors and myokines, which are typically released in response to exercise and thought to be critical in the maintenance of muscle mass and local and systemic metabolism [3, 11, 12, 13]. One of these is irisin, a thermogenic hormone‐like protein recently identified as a PGC‐1α‐dependent and exercise‐responsive myokine [12, 14]. Irisin is likely involved in the control of white adipose tissue browning, insulin sensitivity and maintenance of normal levels of blood glucose and lipids [14, S7]. Irisin is mainly secreted by skeletal muscle tissue, following the cleavage of its precursor, the membrane protein fibronectin type III domain‐containing protein 5 (FNDC‐5). Through the PGC‐1α–irisin–brain‐derived neurotrophic factor (BDNF) axis, irisin mediates numerous positive functions and interorgan communications, including improvement of cognition, learning and memory function [15, 16]. It may also attenuate oxidative stress and neuroinflammation [16, 17, S8]. Recently, it was found that circulating irisin levels were reduced after 60 days of BR [12], while they were unchanged [18] or even increased [19] following 10 or 14 days of BR, respectively. Of note, participants with high serum levels of irisin at the ninth day of BR had the lowest muscle ring finger‐1 (MURF‐1) mRNA levels, and the authors suggested that this indicated that irisin plays a protective role against muscle deterioration in disuse [18]. However, to the best of our knowledge, the relationship between irisin and oxidative stress has not been investigated in disuse.

In recent years, many studies have demonstrated the effectiveness of various exercise‐based countermeasures in preventing or mitigating microgravity‐induced muscle atrophy [20, 21]. For example, whole‐body vibration superimposed on resistive exercise or plyometric training proved beneficial for maintaining muscle power and strength in the lower extremities during prolonged BR [3]. Ballistic strength training and sensorimotor training have also been found effective in improving the maximum isometric voluntary strength, maximum rate of force development and the corresponding neural activation of leg muscles [S2]. Currently, there is a growing interest in the use of artificial gravity (AG)—generated by human centrifugation—to protect the musculoskeletal system. Prompted by early observations that daily 1‐h exposure to AG maintained functional, biochemical and structural homeostasis of skeletal muscle and attenuated muscle atrophy during 21 days of BR [2], the European Space Agency (ESA) and the National Aeronautics and Space Administration (NASA) designed the long‐term (60‐day) BR study AGBRESA. The objective of this study was to assess the effectiveness of a 30‐min daily AG countermeasure to mitigate the adverse physiological effects induced by head‐down tilt (HDT) BR as a ground‐based analogue of spaceflight. Here, as part of the AGBRESA study, we assessed (1) BR‐induced changes in systemic and muscle oxidative stress, (2) their relationships to muscle function and (3) the extent at which human centrifugation attenuated these BR‐induced changes. Moreover, we assessed whether serum levels of irisin and BDNF were related to the extent of systemic oxidant status.

## 2 Methods

### 2.1 Ethical Approval

The AGBRESA study was approved by the Ethics Committee of the North Rhine Medical Association (reference number 2018143) in Düsseldorf, Germany. The AGBRESA study was registered in the German Clinical Trials Register (DRKS‐ID: DRKS00015677). All participants gave written informed consent prior to entering the study. The study adhered to the standards of the Declaration of Helsinki.

### 2.2 Study Design

The main objective of the AGBRESA study was to assess the potential of 30 min daily continuous (cAG) or intermittent (iAG) centrifugation on the adverse impact of 60 days of HDT BR on human physiology [22]. The AGBRESA study was performed in two campaigns between March 2019 and December 2019, under collaboration between ESA, NASA and the German Aerospace Centre (DLR). The study was conducted at the DLR, Cologne, Germany. Each campaign included three experimental phases: (I) a 14‐day baseline data collection (BDC) phase (BDC‐14 through BDC‐1), (II) a 60‐day 6° HDT BR phase (HDT1 through HDT60) and (III) a 14‐day recovery (R+) phase (R + 0 through R + 13) (Figure S1). The AG protocols consisted of a 30‐min daily continuous or a 30‐min intermittent (6 cycles of 5‐min AG separated by 3‐min of rest) exposure to centripetal acceleration by short‐radius centrifugation with the participants in supine position eliciting a centripetal acceleration of 1 Gz at their centre of mass and approximately 2 Gz at their feet (details about the study have been published elsewhere [22]).

### 2.3 Participants

Twenty‐four healthy subjects (16 men, eight women, 33 ± 9 years; 1.75 ± 0.09 m; 74 ± 10 kg) participated in the study and were assigned to one of the following three groups: (1) Control group (BR without AG (*n* = 8)); (2) cAG group (BR plus daily continuous AG (30 min of continuous centrifugation (*n* = 8)); (3) iAG group (BR plus daily intermittent AG (6 × 5 min of centrifugation (*n* = 8)). Participants were pseudorandomly assigned to each group for the first campaign, while for the second campaign, the assignment was designed to demographically balance groups (in terms of men/women distribution (5 m, 3 w), age and body mass of participants), as one man and three women dropped out and were replaced during the second campaign. The new participants started 3 weeks after the other eight participants in the second campaign and completed the entire study protocol as planned [22].

All participants were recruited after rigorous medical, psychological and physiological screening. Details on the subject recruitment process, inclusion and exclusion criteria and the participants' daily routines in the AGBRESA study have been reported elsewhere [22, 23, S9]. Anthropometric data of the AGBRESA participants are shown in Table S1 and are available in [22]. Further details can be found in the guidelines for standardisation of BR studies in the spaceflight context [24].

The data reported in the present study were recorded on the following days: BDC‐5, BDC‐3, BDC‐1, HDT6, HDT55, HDT57, R + 0 and R + 2. Muscle biopsies and blood samples were taken at BDC (baseline), HDT6, HDT55 and HDT57 (Days 6 and 55 or 57 HDT BR, respectively). Data on muscle performance were collected at BDC‐5 and BDC‐3 and at R + 0 and R + 2 (Figure S1).

### 2.4 Lean Mass

Lean mass (whole body) was assessed by dual‐energy X‐ray absorptiometry (DEXA), using the whole‐body scan feature of the Prodigy Full Pro system (GE Healthcare GmbH). Measures were performed before (BDC‐4) and at 60 days of BR (HDT60). Lean mass of both legs was assessed. Data were analysed using the manufacturer's enCORE software (version 16.10.151). These measurements were obtained as part of the BR/core data international standard measures [24].

### 2.5 Maximal Voluntary Contraction (MVC): Isometric Knee Extension and Knee Flexion

The MVC torque was recorded during repeated isometric knee extensions and flexions of the right leg, performed with the knee joints at 80° flexion, and the hip at 90°, using the Isomed 2000 isokinetic dynamometer (D&R Ferstl GmbH, Hemau, Germany), as described previously [23]. Before the test, the participants were familiarised with the equipment and performed a warm‐up consisting of 5 min of light exercise on a cycle ergometer followed by a series of submaximal contractions. The test consisted of three 5‐s repetitions, during which subjects were instructed to contract their muscles ‘as forcefully as possible’ with a progressive force build‐up for each contraction. Each repetition was interposed by 30 s rest. The MVC was recorded 5 days before the start of BR (BDC‐5) and 2 days after the end of the BR period (R + 2). These measurements were obtained as part of the BR/core data international standard measures [24]. All subjects received verbal encouragement during all volitional measurements. The encouragement was given by the operators.

### 2.6 Electrically Evoked Twitch

Maximum twitch force of the knee extensor (KE) and plantar flexor (PF) muscles was evoked by supramaximal electrical stimulation (DS7AH, Welwyn Garden city, United Kingdom), as described in [S10], with the subjects in a seated position on custom‐made knee and ankle ergometers. Electrical pulses were directly delivered over the soleus and vastus lateralis muscle bellies, using large electrodes [S10], and standardised locations relative to specific anatomical landmarks [S10]. The vastus lateralis muscle was stimulated using two large (12.7 × 7.6 cm) self‐adhesive electrodes placed 5–10 cm below the inguinal crease and 5–10 cm above the superior border of the patella over the muscle belly. For the activation of the soleus muscle, the positive electrode was placed above the Achilles tendon, which allows the isolated activation of the soleus; the negative electrode was placed below the heads of the gastrocnemius. During the KE testing, the hip and knee joints were at 90° and 60° of flexion, respectively, whereas for the PF testing, the hip, knee and ankle joints were at 90° [23]. Twitch peak force, electromechanical delay (EMD, i.e. the latency between neural drive to the muscle and start of force development during voluntary contractions [S2]), half relaxation time (HRT), time to peak tension (TTP) and peak rate of force development (%RFD) and relaxation (% RFR) normalised for maximal twitch force (the normalisation for maximal twitch force removes strength as a factor influencing the slope of the force–time curve and provides information on the intrinsic physiological properties of muscles as well as the mechanical properties of the tendon) were collected at baseline (BDC‐3) and on the first day after BR (R + 0).

### 2.7 Submaximal Isometric and Fast Isokinetic Muscle Fatigue Resistance

The isometric muscle fatiguability of KE muscles was determined before the start of BR (BDC‐5) and 2 days after the end of the BR period (R + 2). Participants were asked to sustain 50% MVC maximal voluntary isometric contraction (knee extension) at an 80° knee angle for 90 s. Only in rare cases can someone sustain a 50% MVC over 90 s, and typically, there is a drop in force. The isometric fatigue index was calculated as 100% × final torque (1 s average)/initial torque (1 s average); by applying this formula, a greater FI value indicates lower muscle fatigue, since the final torque is larger than the initial torque [S11, S12]. These measures were part of the ESA's BR core data, and verbal encouragement was provided to the subjects to reach the target torque until the finish time.

The test to determine the isokinetic fatiguability of KE and flexor muscles consisted of 20 repetitions of maximal isokinetic knee extension and flexion at a velocity of 180°/s, with a joint angle ranging from 20° to 95°. The maximum torque value within the first four repetitions and the average of the maximum torque values of the last three repetitions were used for analyses. The isokinetic fatigue index was calculated as 100% × Torque_end_/Torque_start_, where a greater FI value indicates lower muscle fatigue, since the final torque is larger than the initial torque [S11, S12]. The isokinetic test was performed 5 days before the start of BR (BDC‐5) and 2 days after the end of the BR period (R + 2). Knee extension and flexion torque data were recorded with the Isomed 2000 system (D&R Ferstl GmbH, Hemau, Germany). These measures were obtained as part of the international standard measures [24].

### 2.8 Oxidant and Antioxidant Systemic Status

To assess the systemic oxidant and antioxidant status during the early and late disuse periods, analyses were performed on serum samples of venous blood collected before (BDC‐1), after 6 days (HDT6), and at the end of BR (HDT57). The venous blood samples were obtained after an overnight fast, centrifuged, and the serum samples were stored at −80°C. Analyses were performed in triplicate for all three experimental time points (BDC‐1, HDT6, HDT57) in all 24 subjects.

Serum levels of 8‐iso‐prostaglandin F2α (8‐iso‐PGF2α, a marker of lipid peroxidation) were measured using the direct 8‐iso‐PGF2α ELISA detection assay (Enzo Life Sciences, Farmingdale, NY, United States) as per manufacturer recommendations. The end‐point optical density was measured at 405 nm, with correction between 570 and 590 nm, using a microplate reader (FLUOstar Omega microplate reader, BMG LABTECH, Ortenberg, Germany). To measure total 8‐iso‐PGF2α (sum of free isoprostane and its esterified form), 400 μL of serum was treated with 10 N NaOH, followed by neutralisation with 37% HCl to induce hydrolysis of the lipoprotein or phospholipid‐coupled 8‐iso‐PGF2α before the start of the assay. Activity levels of serum superoxide dismutases (including copper‐zinc superoxide dismutase (Cu/ZnSOD, SOD1), manganese superoxide dismutase (MnSOD, SOD2) and extracellular superoxide dismutase (EC‐SOD, SOD3))—key enzymes in the antioxidant defence system—were determined using the SOD Colorimetric activity kit (Life Technologies, Thermo Fisher Scientific Inc., Carlsbad, United States), as per manufacturer recommendations. Serum samples were diluted 1:5 in 1X assay buffer before the start of the assay. To avoid haemoglobin interference, the blanking of the plate was run before adding the chromogenic detection reagent. The final obtained activity value was normalised for the total protein concentration in each serum sample.

Blood homocysteine, lactate dehydrogenase (LDH) and urine 3‐methylhistidine (an index of muscle protein breakdown) levels were obtained as part of the international standard measures on BDC‐3 and HDT60/R + 0 [24].

### 2.9 Quantification of Serum Irisin Levels

The serum concentration of irisin was determined in duplicate for all three experimental time points (BDC‐1, HDT6, HDT57) in all 24 subjects, with an ELISA immunoassay kit (Cat# AG‐45A‐0046YEK‐KI01 AdipoGen Life Sciences, Füllinsdorf, Switzerland), following the procedure recommended by the manufacturer.

Serum BDNF and C‐terminal agrin fragment (CAF) data used for the correlation analysis with irisin and neuromuscular protection parameters in the present paper have been published elsewhere [25].

### 2.10 Quantification of Serum and Muscle Total Protein Concentrations

Total protein concentrations in homogenates and in serum samples were determined by the colorimetric BCA‐protein assay (Pierce BCA Protein Assay Kit, Life Technologies, Carlsbad, United States), with absorbance measured at 562 nm using a microplate reader (BioTek Synergy H1, Agilent, Santa Clara, CA, United States). Analyses were performed in triplicate.

### 2.11 Muscle Biopsies

Muscle biopsies from the vastus lateralis muscle were collected before BR (BDC‐1), after 6 days of BR (HDT6) and at the end of the BR at day 55 (HDT55) using a biopsy rongeur (4‐mm diameter), following skin disinfection and local anaesthesia with lidocaine, before incision of the skin and fascia. Biopsies were frozen in liquid nitrogen and stored at −80°C until analysis. Due to the low amount of tissue, the sample size varied per experiment. Overall, molecular and biochemical analyses were completed on muscle samples from 19 subjects where the complete set of biopsies was available (BDC‐1; HDT6; HDT55) (control, six subjects; AG, 13 subjects) and five subjects had an incomplete set of biopsies (control, two subjects; AG, three subjects).

### 2.12 Quantification of Muscle Protein Carbonyl Concentration

The levels of carbonylated proteins in biopsies of the vastus lateralis muscle were assessed using a commercial ELISA detection kit (Protein Carbonyl ELISA kit, Cat# ALX‐850‐312‐KI01, ENZO Life Sciences, United States), as recommended by the manufacturer. Before the assay, the muscle biopsies were cleaned of any traces of blood and then cut into small pieces. Total protein was then extracted from around 5 to 10 mg of frozen muscle samples by homogenisation in ice‐cold phosphate‐buffered saline ((Na_2_HPO_4_ • 2 H_2_O (8.1 mmol/L), KH_2_PO_4_ (1.76 mmol/L), NaCl (137 mmol/L) and KCl (2.7 mmol/L), pH = 7.4) without detergents to avoid interference with the ELISA plate coating. The homogenate was left on ice for 20 min and finally centrifuged at 10 000 RPM for 5 min at 4°C to have a supernatant with proteins. Before performing the ELISA, 30 μg of protein extract was chemically derivatised with 200 μL dinitrophenylhydrazine and diluted following the specifications given in the kit. As specified by the manufacturer, the assay is set up to saturate each ELISA well with about 1 μg of derivatised protein. Sample absorbances were read at 450 nm (FLUOstar Omega microplate reader (BMG LABTECH) Ortenberg, Germany) immediately after stopping the reaction. Analyses were performed in quadruplicate.

### 2.13 Quantification of Activity Levels of Muscle Superoxide Dismutases

Activity levels of muscle superoxide dismutases were determined using the SOD Colorimetric activity assay kit (Cat# EIASODC, Life Technologies, Thermo Fisher Scientific Inc., Carlsbad, United States), as recommended by the manufacturer. Total proteins were extracted as described above. The final activity value was normalised for the total protein concentration in each muscle sample. Analyses were performed in triplicate.

### 2.14 Real‐Time RT‐PCR

Total RNAs were extracted from the biopsy samples (5–20 mg muscle tissue) using the miRNeasy Micro Kit (Qiagen, Hilden, Germany). Total RNA reverse‐transcription was performed with SuperScript IV First Strand System with EZDNASE (Invitrogen, Carlsbad, CA, United States) in the presence of random primers. Analyses were then performed by real‐time PCR (CFX Connect Real Time PCR detection system with Maestro software, Version 4.1.2433.1219 by Biorad, Hercules, CA, United States) with the SYBR Green PCR kit (Invitrogen, Carlsbad, CA, United States). Relative quantities were calculated by the ΔCTs (2^−ΔCT^) method. The geometric mean of threshold cycle values of Glyceraldehyde 3‐phosphate dehydrogenase (GAPDH) and β‐2 microglobulin reference genes was used to normalise the data. The PCR parameters were initial denaturation at 95°C for 10 min followed by 40 cycles of 15 s at 95°C and 60 s at the corresponding annealing temperature: 59°C for 8‐oxoguanine glycosylase (OGG1), haeme‐oxygenase (HO‐1) and β‐2 microglobulin; 56°C for p66^(ShcA)^; 61°C for muscle RING‐finger protein‐1 (MURF‐1), Atrogin‐1 and GAPDH; 58°C for nuclear factor erythroid 2‐related factor 2 (NRF‐2); 60°C for Sirtuin‐1 and Myostatin, followed by melting curves for the acquisition of fluorescence signals. The forward and reverse primers used for RT‐PCR MURF‐1, Atrogin‐1 and NRF‐2 were the same as those in [S13, S14]. Forward and reverse primer sequences are reported in Table S2. Analyses were performed in duplicate.

## 3 Statistics

Data were analysed with GraphPad ver.9.4. The Shapiro Wilk's test was used to determine whether the data were normally distributed. Repeated‐measures 2‐way ANOVA was applied using time (BDC‐1, HDT6, HDT57) or time (HDT6, HDT57) ratio % on BDC as within and group (controls, AG) as between‐subject factors. A two‐way linear mixed‐effect model was applied in the case of missing data. In addition to main effects, two‐way interactions were also analysed. The analyses of variance were followed by Šidák correction for multiple comparisons when significant group × time interactions were found. Similar to our previous study [26], there were no significant group × time interactions, and therefore, the participants from the cAG and iAG groups were pooled (AG group) for further analysis. To analyse sex differences in response to BR, data from the three groups were pooled to allow a comparison between the eight women and 16 men. Data were analysed by a two‐way linear mixed‐effect model followed by Šidák correction for multiple comparisons when significant time × sex interactions were found.

To compare changes in strength irrespective of changes in body mass and lean mass, statistical analyses were also performed by normalising the MCV values for body mass and lean mass of the right leg.

CAF and irisin tertile groups were analysed by one‐way ANOVA for multiple comparisons or the Kruskal–Wallis test in case of nonparametric data distribution.

Spearman's (for data with nonparametric distribution) or Pearson's (for data with parametric distribution) linear regression analysis was used to identify significant correlations between parameters. Data are presented as means ± SEM or means ± *SD*. Differences were considered significant for *p* < 0.05. For the AGBRESA study, the estimated required sample size in each group (*n* = 8) was based on data obtained in previous BR studies to obtain an effect size of 0.5, an alpha error of 0.05 and a power of 0.8 [23].

## 4 Results

No significant differences in the effects of BR were found between cAG and iAG, and therefore, for further analysis, the data from the cAG and iAG groups were pooled and presented as the AG group.

### 4.1 Effect of BR and AG on Maximal Voluntary Isometric Torque (MVC) of KE and Flexor Muscles

At baseline, the MVC torque was lower in women than in men (Table 1; *p* < 0.0001). There was no significant time × sex interaction; nonetheless, the drop (%Δ change from pre‐BR) in MVC torque was greater in women than in men (Table 1). A significant time × sex interaction was found following normalisation of MVC torque of knee flexors for body mass (*p* = 0.035) and leg lean mass (*p* = 0.011). In accordance with previous findings [23], 60 days of BR induced a reduction in maximal isometric torque of both KE and knee flexor muscles (*p* < 0.001), irrespective of AG (Table 1). The time × group interactions (*p* < 0.01) were reflected by a larger absolute reduction in MVC in the control than in the AG group, but the % decline did not differ significantly between the two groups (Figure S2 **).**

**TABLE 1:** Maximal voluntary isometric torque (MVC) of knee extensor and flexor muscles normalised for body mass and leg lean mass.

Panel A Body mass, lean mass and leg lean mass								
	AG		Controls		*p*		*p*	*p*
	Pre‐BR	Post‐BR	Pre‐BR	Post‐BR	Post‐BR versus Pre‐BR	Time * group interaction	Group effect	Δ% AG versus controls
Body mass (kg)	71.3 ± 1.9	71.2 ± 1.9	79.3 ± 4.5	78.4 ± 4.6	0.015	ns	ns	ns
Δ%	−0.3 ± 0.3%		−1.3 ± 0.5%					
Body total lean mass (kg)	50.9 ± 2.5	48.0 ± 2.5	56.0 ± 2.8	52.8 ± 2.9	< 0.001	ns	ns	ns
Δ%	−6.1 ± 0.7		−5.8 ± 0.6					
Leg lean mass (kg)	8.7 ± 0.4	7.7 ± 0.4	9.6 ± 0.5	8.4 ± 0.5	< 0.001	ns	ns	ns
Δ%	−12.2 ± 1.0%		−12.6 ± 1.7%					

	Men		Women		*p*		*p*	*p*
	Pre‐BR	Post‐BR	Pre‐BR	Post‐BR	Post‐BR versus pre‐BR	Time * sex interaction	Sex effect	Δ% men versus women
Body mass (kg)	78.2 ± 1.8	78.1 ± 1.8	65.6 ± 3.4	64.5 ± 3.2	0.005	0.010	< 0.001	0.005
Δ%	−0.1 ± 0.3		−1.7 ± 0.5					
Body total lean mass (kg)	58.5 ± 1.0	55.5 ± 1.1	40.9 ± 1.9	37.8 ± 1.8	< 0.001	ns	< 0.001	0.012
Δ%	−5.2 ± 0.6		−7.6 ± 0.5					
Leg lean mass (kg)	9.9 ± 0.2	8.9 ± 0.2	7.1 ± 0.4	5.9 ± 0.3	< 0.001	ns	< 0.001	< 0.001
Δ%	−10.4 ± 0.7		−16.2 ± 1.3					

Panel B Knee extension								
	AG		Controls		*p*		*p*	*p*
	Pre‐BR	Post‐BR	Pre‐BR	Post‐BR	Post‐BR versus pre‐BR	Time * group interaction	Group effect	Δ% AG versus controls
MVC (Nm)	228.1 ± 19.5	169.4 ± 17.8	300.8 ± 26.8	197.9 ± 25.9	< 0.001	0.0005	ns	ns
Δ%	−26.9 ± 3.0%		−36.0 ± 3.4%					
MVC per body mass (Nm/kg)	3.2 ± 0.2	2.3 ± 0.2	3.8 ± 0.3	2.5 ± 0.3	< 0.001	0.005	ns	ns
Δ%	−26.8 ± 2.9%		−35.2 ± 3.3%					
MVC per leg lean mass (Nm/kg)	25.8 ± 1.3	21.4 ± 1.9	31.0 ± 1.4	22.9 ± 2.2	< 0.001	0.008	ns	ns
Δ%	−16.9 ± 3.1%		−27.0 ± 2.9%					

	Men		Women		*p*		*p*	*p*
	Pre‐BR	Post‐BR	Pre‐BR	Post‐BR	Post‐BR versus pre‐BR	Time * sex interaction	Sex effect	Δ% men versus women
MVC (Nm)	296.0 ± 15.2	220.1 ± 12.1	165.1 ± 15.0	96.5 ± 4.8	< 0.001	ns	< 0.001	0.002
Δ%	−25.1 ± 2.4%		−39.7 ± 3.5%					
MVC per body mass (Nm/kg)	3.8 ± 0.2	2.8 ± 0.2	2.5 ± 0.2	1.5 ± 0.1	< 0.001	ns	< 0.001	0.003
Δ%	−25.0 ± 2.4%		−38.7 ± 3.3%					
MVC per leg lean mass (Nm/kg)	29.7 ± 1.3	24.7 ± 1.2	23.1 ± 1.5	16.3 ± 0.7	< 0.001	ns	< 0.001	0.023
Δ%	−16.4 ± 2.6%		−27.9 ± 4.3%					

Panel C Knee flexion								
	AG		Controls		*p*		*p*	*p*
	Pre‐BR	Post‐BR	Pre‐BR	Post‐BR	Post‐BR versus pre‐BR	Time * group interaction	Group effect	Δ% AG versus controls
MVC (Nm)	102.0 ± 7.7	90.2 ± 9.1	114.1 ± 8.3	87.0 ± 8.0	< 0.001	0.026	ns	ns
Δ%	−14.7 ± 4.7%		−24.0 ± 4.4%					
MVC per body mass (Nm/kg)	1.4 ± 0.1	1.3 ± 0.1	1.5 ± 0.1	1.1 ± 0.1	< 0.001	ns	ns	ns
Δ%	−14.5 ± 4.6%		−23.1 ± 4.1%					
MVC per leg lean mass (Nm/kg)	11.7 ± 0.6	11.5 ± 0.8	12.0 ± 0.7	10.3 ± 0.7	0.029	ns	ns	ns
Δ%	−3.2 ± 4.8%		−13.4 ± 3.8%					

	Men		Women		*p*		*p*	*p*
	Pre‐BR	Post‐BR	Pre‐BR	Post‐BR	Post‐BR versus pre‐BR	Time * sex interaction	Sex effect	Δ% men versus women
MVC (Nm)	120.1 ± 4.7	107.1 ± 4.7	77.8 ± 8.8	53.3 ± 6.9	< 0.001	ns	< 0.001	0.0004
Δ%	−10.2 ± 3.5%		−32.9 ± 4.5%					
MVC per body mass (Nm/kg)	1.6 ± 0.1	1.4 ± 0.1	1.2 ± 0.1	0.8 ± 0.1	< 0.001	0.035	< 0.001	< 0.001
Δ%	−10.2 ± 3.4%		−31.7 ± 4.6%					
MVC per leg lean mass (Nm/kg)	12.2 ± 0.5	12.1 ± 0.6	11.0 ± 1.1	8.9 ± 1.0	0.001	0.011	0.038	0.004
Δ%	0.1 ± 3.7%		−20.1 ± 5.0%					

### 4.2 Effect of BR and AG on KE and PF Twitch Contractile Properties

Following BR, and irrespective of AG, the EMD of KEs was elongated in women but not in men (w: BDC, 12.2 ± 0.5 ms; R + 0, 17.4 ± 1.5 ms; m: BDC, 11.9 ± 0.7 ms; R + 0, 11.4 ± 0.8 ms, sex × time interaction, *p* = 0.0041). No significant changes or differences in EMD between the sex responses were observed for the PFs (w: BDC, 10.8 ± 0.5 ms; R + 0, 11.3 ± 0.9 ms; m: BDC, 10.2 ± 0.4 ms; R + 0, 10.7 ± 0.4 ms). No significant sex × time interactions for any of the other parameters of KE or PF were found.

Overall, BR caused a significant loss in the electrically evoked twitch peak torque in both KE and PF muscles. The loss in peak twitch torque of the KE did not differ significantly between the Control and AG groups (no significant time × group interaction; Table 2), but the loss of twitch peak torque of PF was less pronounced in the AG (25%) than in the Control (48%) group (time × group interaction, *p* = 0.020; Table 2), suggesting a protective effect of centrifugation on PF evoked twitch torque.

**TABLE 2:** Effect of BR and AG on twitch contractile properties of knee extensor and plantar flexor muscles.

Knee extensor muscles						
	AG		Controls		*p*	
	BDC‐3	R + 0	BDC‐3	R + 0	R + 0 versus BDC‐3	Time * group interaction
Peak force (N)	102.0 ± 7.4	73.3 ± 5.6	113.5 ± 11.3	80.8 ± 7.5	< 0.001	ns
EMD (ms)	12.1 ± 0.7	14.2 ± 1.2	11.8 ± 0.4	12.9 ± 0.3	ns	ns
%RFD (%(force/s)/peak twitch force)	2499 ± 42	2699 ± 41	2423 ± 111	2659 ± 84	< 0.001	ns
%RFR (%(force/s)/peak twitch force)	−1119 ± 63	−1590 ± 101	−1039 ± 103	−1904 ± 151	< 0.001	0.030
HRT (ms)	75.7 ± 4.4	51.3 ± 5.1	88.5 ± 5.0	39.5 ± 3.6	< 0.001	0.009
TTP (ms)	72.3 ± 0.9	69.7 ± 0.8	75.5 ± 2.7	72.9 ± 1.7	0.016	ns

Plantar flexor muscles						
	AG		Controls		*p*	
	BDC‐3	R + 0	BDC‐3	R + 0	R + 0 versus BDC‐3	Time * group interaction

Peak force (N)	109.7 ± 8.6	82.2 ± 6.8	103.8 ± 9.3	54.1 ± 6.7	< 0.0001	0.020
EMD (ms)	10.3 ± 0.4	10.7 ± 0.5	10.5 ± 0.4	11.1 ± 0.7	ns	ns
%RFD (%(force/s)/peak twitch force)	1746 ± 49	1785 ± 59	1727 ± 58	1800 ± 28	ns	ns
%RFR (%(force/s)/peak twitch force)	−1002 ± 36	−1163 ± 59	−1185 ± 96	−1334 ± 41	0.003	ns
HRT (ms)	83.2 ± 3.2	73.0 ± 3.2	70.2 ± 4.7	58.3 ± 2.0	0.001	ns
TTP (ms)	115.3 ± 4.8	109.2 ± 5.0	114.7 ± 4.6	97.2 ± 4.2	0.002	ns

BR increased the evoked twitch speed of KE, as shown by the reduction in HRT, the reduction of the TTP and the increased % of the peak rate of torque development (RFD) (Table 2; Figure S3). Furthermore, the reduction in HRT and the increase in % of the peak rate of relaxation (RFR) were greater in the control than in the AG group (time × group interaction, *p* = 0.009 and *p* = 0.030; Table 2; Figure S3), indicating that AG attenuated the reduction in HRT and the increase in %RFR in the KE. Irrespective of AG, BR also caused a decrement in the HRT (no significant time × group interaction) in the PF, while the %RFD was not significantly affected (Table 2).

### 4.3 Effect of BR and AG on Isometric and Isokinetic Muscle Fatigue of KE Muscles

**FIGURE 1:**
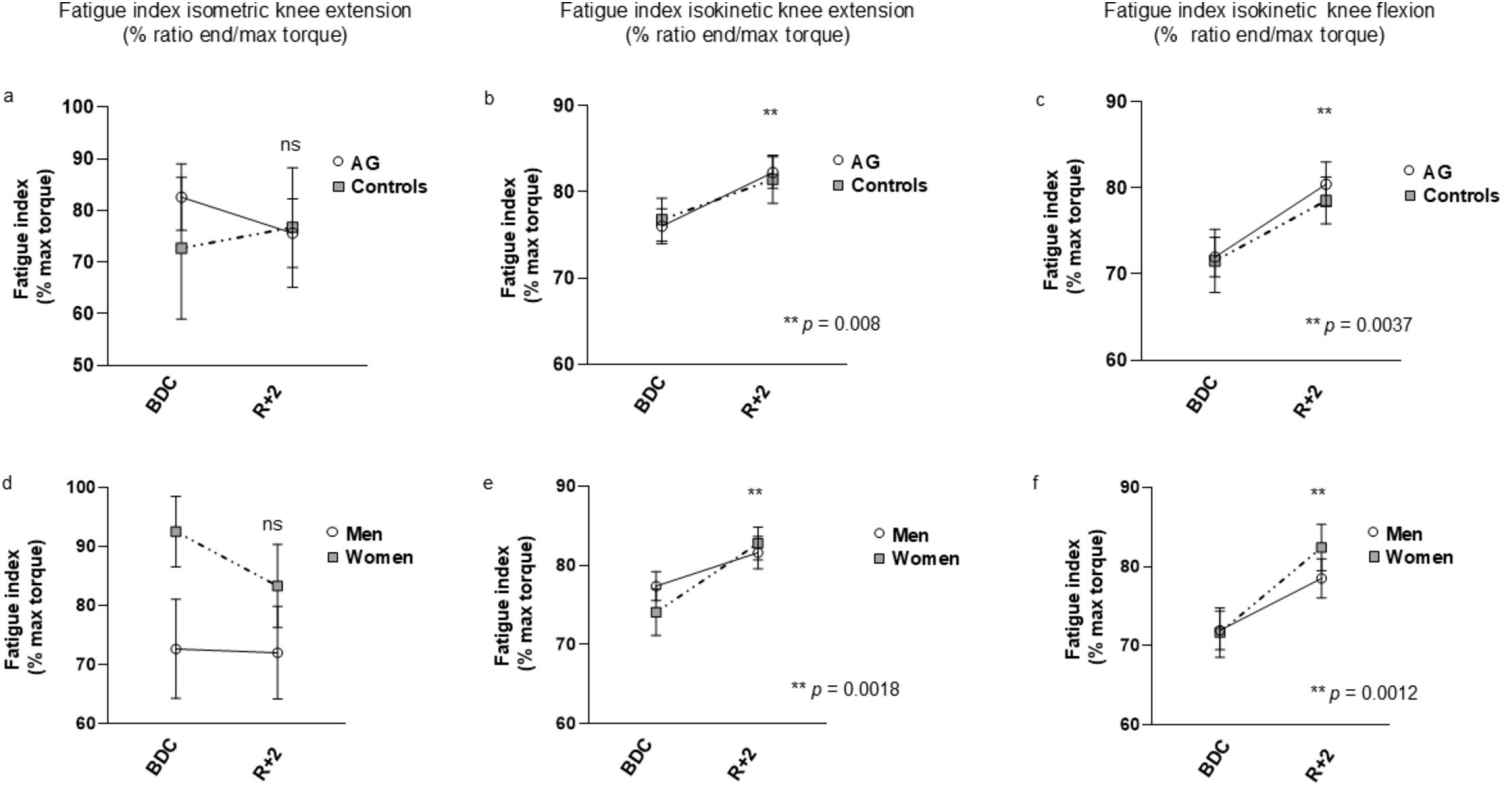
Submaximal isometric and fast isokinetic muscle fatigue before and after bed rest. (a) Submaximal isometric fatigue index of knee extensor muscles before and after bed rest, respectively in the AG and the Control groups and (d) in women and men. Isokinetic fatigue index of knee extensors (b) and flexors (c) before and after bed rest, respectively in the AG and Control groups (b, c) and (e, f) in women and men. BDC: baseline data collected at 5 days before the start of bed rest; R + 2, data collected 2 days after bed rest; Controls, bed rest group without intervention; AG: cAG and iAG pooled subjects. Data are presented as mean ± SEM Differences were considered significant at *p* < 0.05.

Irrespective of sex or AG, we did not find any significant impact of BR on the development of muscle fatigue following a 90‐s sustained isometric knee extension at 50% MVC (Figure 1). A different response to BR was observed following a series of maximal voluntary isokinetic contractions at 180 deg/s, where, after BR, the fatigue index was higher compared with that before BR, indicating less fatigue development following BR (Figure 1).

### 4.4 BR and Oxidant/Antioxidant Responses

**FIGURE 2:**
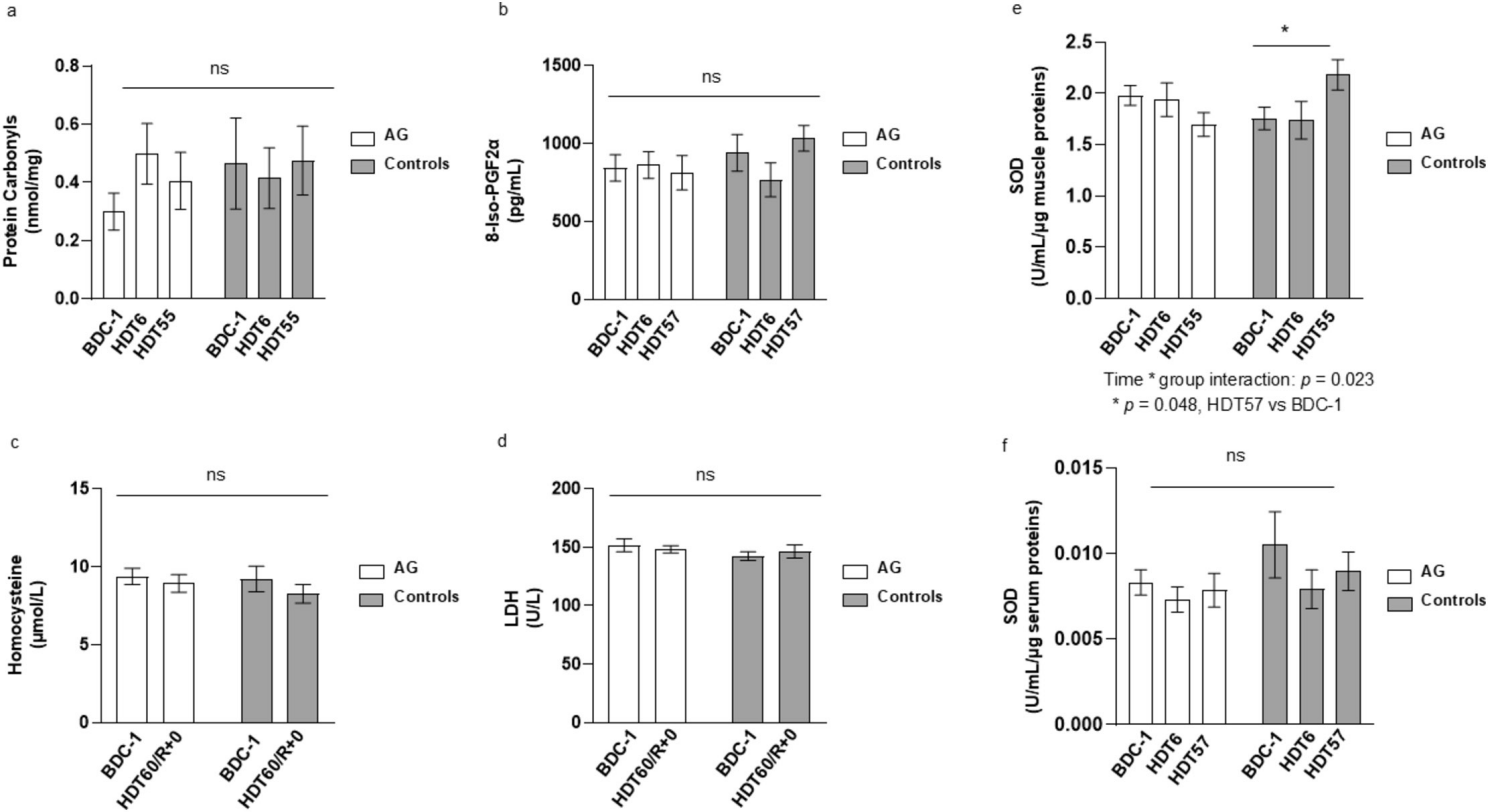
Effect of BR and AG on muscle protein carbonyl contents (a), oxidative stress‐related circulating biomarker (b–d), and superoxide dismutase activity in vastus lateralis muscle biopsies (e) and serum (f). BDC‐1: baseline data collected 1 day before the start of bed rest; HDT6, 6 days of bed rest; HDT55, 55 days of bed rest; HDT57, 57 days of bed rest; HDT60/R + 0, data collected at the end of the bed rest period. Controls, bed rest group without intervention; AG: cAG and iAG pooled subjects. LDH, lactate dehydrogenase. SOD, superoxide dismutase. Data are presented as mean ± SEM. Differences were considered significant at *p* < 0.05.

Oxidative modifications of myofibrillar proteins may contribute to the reduced force‐generating capacity of the muscle [5, S15]. To explore this, we assessed the total muscle carbonyl contents in muscle biopsies obtained before and at various BR time points (Figure 2a), and transcript abundance of key proteins involved in the modulation of oxidant/antioxidant cell status and cell protection (Table 3). BR, with or without AG, did not induce a significant change in muscle carbonyl contents (Figure 2a). However, BR increased the mRNA levels of proteins involved either in the generation of free radicals and mitochondrial damage (p66^(ShcA)^) or in cell‐protection and mitochondrial metabolism, such as NRF‐2 and OGG1. The increase was significant at the late phase (HDT55) but not in the early phase of BR (HDT6) (Table 3).

**TABLE 3:** Muscle mRNA levels of key proteins involved in the modulation of oxidant/antioxidant cell status and cell protection.

Muscle mRNA levels (arbitrary units * 10.000)						
		BDC‐1	HDT6	HDT55	Time effect	Time * group interaction
PGC‐1α	AG	74 ± 8	52 ± 7	68 ± 6	ns	ns
	Controls	66 ± 8	72 ± 1	69 ± 1		
FNDC5	AG	550 ± 81	490 ± 66	540 ± 51	ns	ns
	Controls	510 ± 64	590 ± 51	540 ± 56		
MuRF‐1	AG	160 ± 12	170 ± 17	170 ± 24	ns	ns
	Controls	140 ± 13	240 ± 42	270 ± 11		
Atrogin‐1	AG	570 ± 43	600 ± 65	570 ± 37	ns	ns
	Controls	570 ± 45	820 ± 15	520 ± 32		
Myostatin	AG	57 ± 10	120 ± 24^b^	140 ± 22^a^	*p* = 0.001	ns
	Controls	56 ± 9	120 ± 34^b^	140 ± 30^a^		
OGG1	AG	0.82 ± 0.10	0.79 ± 0.08	1.50 ± 0.29^a,c^	*p* = 0.014	ns
	Controls	0.78 ± 0.12	0.78 ± 0.14	1.10 ± 0.20^a,c^		
p66^(ShcA)^	AG	21.0 ± 2.2	19.2 ± 2.5	34.0 ± 3.4^a,c^	*p* = 0.016	ns
	Controls	19.0 ± 2.0	25.3 ± 1.2	24.7 ± 5.2^a,c^		
Sirt‐1	AG	6.3 ± 0.6	12.0 ± 3.7	9.3 ± 1.1	*ns*	ns
	Controls	6.2 ± 0.7	7.9 ± 0.8	8.0 ± 1.4		
NRF‐2	AG	71 ± 6	82 ± 14	120 ± 9^a,c^	*p* = 0.002	ns
	Controls	63 ± 5	86 ± 22	110 ± 13^a,c^		
Haeme‐oxygenase	AG	14 ± 2	65 ± 4	27 ± 9	ns	ns
	Controls	17 ± 2	16 ± 3	17 ± 4		

*Note:* a, HDT55 versus BDC‐1; b, HDT6 versus BDC‐1; c, HDT55 versus HDT6.

We observed no significant change in mRNA levels of Sirt‐1, a deacetylase that is involved in maintaining redox homeostasis [S16], but there was an increase of SOD activity (one of the key antioxidant enzymes) in muscle biopsies of control subjects at the end of BR, while no significant changes were observed in AG groups (time × group interaction: *p* = 0.023; Figure 2e). Overall, these data suggest that muscle cell protection was not hampered during the 60 days of BR.

These changes were accompanied by a significant increase in urine 3‐methylhistidine levels (Figure S4) and muscle myostatin (a myokine which upregulation is associated with muscle wasting) mRNA levels, which was detectable already after 6 days of BR (Table 3). Furthermore, although BR increased the mRNA levels of some oxidant (p66^(ShcA)^) and antioxidant (NRF‐1, OGG1 and SOD) biomarkers in muscle tissue, there were no significant changes in the systemic levels of the oxidant/antioxidant status, as indicated by the absence of significant alterations in the circulating levels of some oxidative stress‐related biomarkers (Figure 2b–d) and in serum SOD activity (Figure 2f). Nonetheless, we found a negative relationship between the serum levels of 8‐iso‐PGF2α and KE muscle isokinetic fatigue index, both in all data time points combined (*r* = −0.376; *p* = 0.0083; Figure S5a) and following BR (*r* = −0.438; *p* = 0.032; Figure S5b).

### 4.5 Blood Irisin and the PGC‐1alpha/BDNF Axis

**FIGURE 3:**
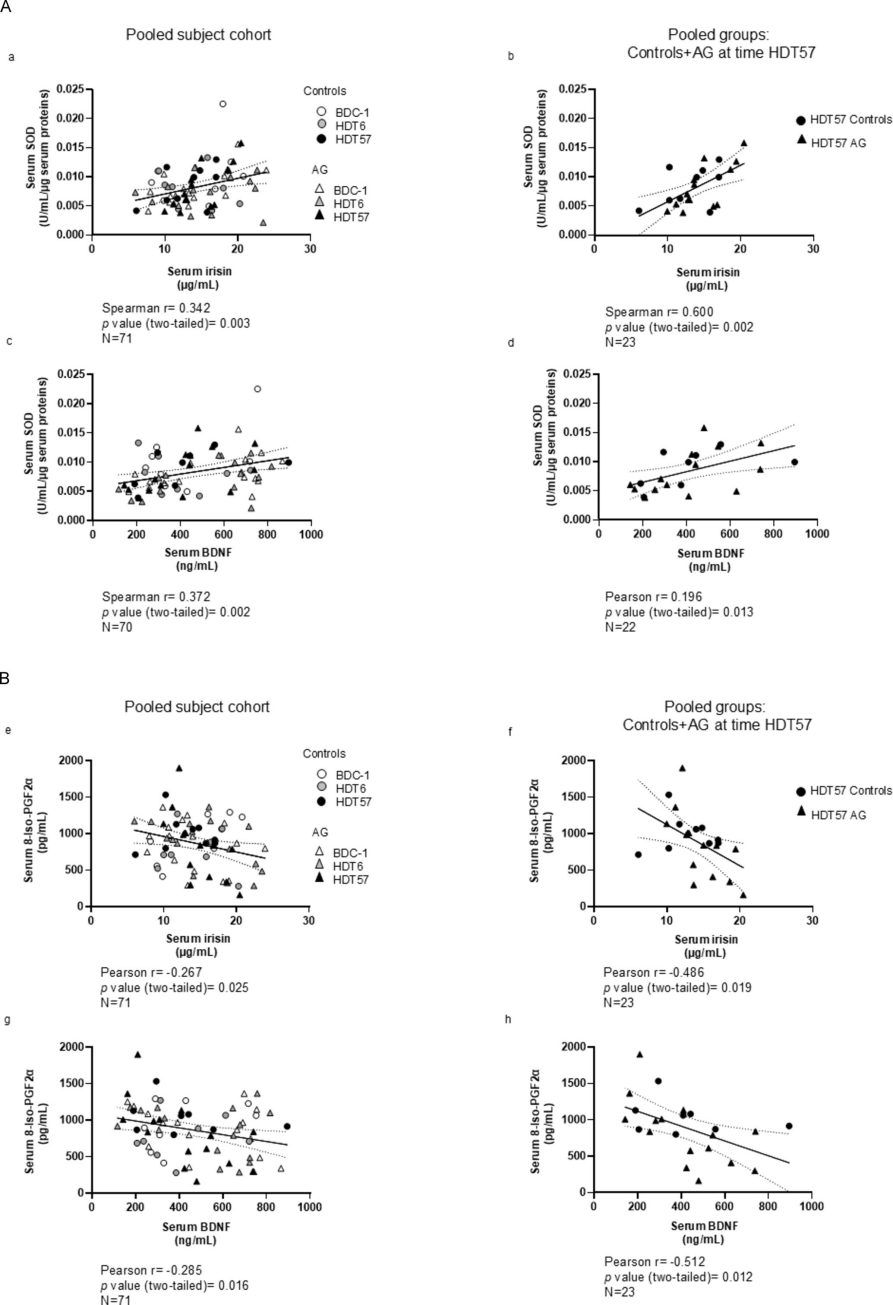
Relationship between serum irisin, brain‐derived neurotrophic factor (BDNF), antioxidant (Panel A) and oxidant (Panel B) biomarkers. (a,c,e,g) pooled experimental time points (BDC‐1 + HDT6 + HDT57); (b, d, f, h) data collected at the end of the bed rest period (HDT57). BDC: baseline data collected at 1 day before the start of bed rest; AG: cAG and iAG pooled subjects. HDT6, 6 days of bed rest, HDT57, 57 days of bed rest. Relationships were considered significant at *p* < 0.05.

BR or daily AG did neither significantly affect irisin serum levels (Figure S6) nor muscle mRNA levels of its precursor (FNDC5) and activator (PGC‐1α) (Table 3). Serum irisin concentrations positively correlated with serum levels of BDNF, also following BR (Figure S6). Irisin and BDNF positively correlated with serum SOD activity (Figure 3A) and inversely correlated with the serum levels of 8‐iso‐PGF2α, also following BR (Figure 3B).

Since the CAF serum concentration showed a high variability before BR (mean ± SEM: 242 ± 94 (SD: 461) pg/mL) and during BR (HDT6: 225 ± 80 (SD: 393) pg/mL; HDT57: 194 ± 59 (SD: 290) pg/mL) [25], we stratified the subject cohort in subgroups according to serum CAF tertile levels to analyse the relationship between CAF and irisin, oxidative stress and parameters of neuromuscular protection during the course of BR. In addition, we stratified the subject cohort according to serum irisin tertiles.

At the end of BR, subjects in the highest CAF tertile showed the lowest irisin and SOD serum levels, and (although not significant, *p* = 0.051) BDNF levels (Table 4, Panel A). Consistent with the correlation analysis, at the end of BR, the subjects in the highest irisin tertile group showed the highest BDNF and lowest 8‐iso‐PGF2α and CAF levels (Table 4, Panel B). A similar distribution was found by pooling all experimental times points (Table 4, Panel C). Interestingly, such a potential irisin association with CAF was found before and after 6 days of BR (Table S3).

**TABLE 4:** Distribution of mean serum concentrations of biomarkers by tertiles of serum C‐terminal agrin fragment (CAF) (Panel A) and serum irisin concentration (Panels B and C).

Panel A HDT57 (pooled subject cohort)				
Serum CAF tertiles (pg/mL)				
	First CAF tertile (36–44 pg/mL)	Second CAF tertile (49–83 pg/mL)	Third CAF tertile (87–1090 pg/mL)	*p*
	Mean ± SEM	Mean ± SEM	Mean ± SEM	
CAF (pg/mL)	40 ± 1 (*n* = 8)	58 ± 5^b^ (*n* = 8)	484 ± 128^a,c^ (*n* = 8)	^a^ *p* < 0.001; ^b c^ *p* = 0.023
Irisin (μg/mL)	17.3 ± 0.9 (*n* = 7)	12.7 ± 1.2^b^ (*n* = 8)	12.5 ± 0.8^a^ (*n* = 8)	^a^ *p* = 0.009; ^b^ *p* = 0.001
8‐iso‐PGF 2α (pg/mL)	642 ± 111 (*n* = 8)	937 ± 87 (*n* = 8)	1079 ± 170 (*n* = 8)	ns
SOD (U/mL/μg serum proteins)	0.0111 ± 0.0012 (*n* = 7)	0.0071 ± 0.0010 (*n* = 8)	0.0069 ± 0.0011^a^ (*n* = 8)	^a^ *p* = 0.046
BDNF (ng/mL)	581 ± 59 (*n* = 8)	291 ± 58^b^ (*n* = 7)	373 ± 60 (*n* = 8)	^b^ *p* = 0.008

Panel B HDT57 (pooled subject cohort)				
Serum irisin tertiles (μg/mL)				
	First irisin tertile (6.07–12.67 μg/mL)	Second irisin tertile (12.81–15.78 μg/mL)	Third irisin tertile (16.27–20.45 μg/mL)	*p*
	Mean ± SEM	Mean ± SEM	Mean ± SEM	
CAF (pg/mL)	333 ± 143 (*n* = 8)	206 ± 90 (*n* = 8)	42 ± 2^a,b^ (*n* = 7)	^a^ *p* = 0.004; ^b^ *p* = 0.030
Irisin (μg/mL)	10.5 ± 0.7 (*n* = 8)	14.1 ± 0.4^a^ (*n* = 8)	17.9 ± 0.6^a,b^ (*n* = 7)	^a,^ *p* < 0.001; ^b^ *p* < 0.01
				
8‐iso‐PGF 2α (pg/mL)	1196 ± 139 (*n* = 8)	844 ± 97 (*n* = 8)	619 ± 115^a^ (*n* = 7)	^a^ *p* = 0.006
SOD (U/mL/μg serum proteins)	0.0060 ± 0.0010 (*n* = 8)	0.0085 ± 0.0010 (*n* = 8)	0.0103 ± 0.0015 (*n* = 7)	ns
BDNF (ng/mL)	275 ± 35 (*n* = 8)	429 ± 78 (*n* = 8)	541 ± 74^a^ (*n* = 7)	^a^ *p* = 0.033

Panel C Pooled experimental times (BDC‐1, HDT6, HDT57)				
Serum irisin tertiles (μg/mL)				
	First irisin tertile (5.93–11.87 μg/mL)	Second irisin tertile (12.10–16.44 μg/mL)	Third irisin tertile (16.79–23.91 μg/mL)	
	Mean ± SEM	Mean ± SEM	Mean ± SEM	
CAF (pg/mL)	275.3 ± 91.5 (*n* = 24)	336.1 ± 92.1 (*n* = 24)	50.4 ± 5.1^a,b^ (*n* = 23)	^a^ *p* < 0.001; ^b,^ *p* < 0.001
Irisin (μg/mL)	9.77 ± 0.34 (*n* = 24)	14.31 ± 0.28^a^ (*n* = 24)	19.56 ± 0.44^a,b^ (*n* = 23)	^a^ *p* = 0.003; ^b^ *p* < 0.001
8‐iso‐PGF 2α (pg/mL)	942 ± 59 (*n* = 24)	926 ± 78 (*n* = 24)	729 ± 72 (*n* = 23)	ns
SOD (U/mL/μg serum proteins)	0.0070 ± 0.0005 (*n* = 24)	0.0074 ± 0.0006 (*n* = 24)	0.0104 ± 0.0009^a,b^ (*n* = 23)	^a^ *p* = 0.002; ^b^ *p* = 0.005
BDNF (ng/mL)	388 ± 41 (*n* = 23)	420 ± 46 (*n* = 24)	584 ± 38^a,b^ (*n* = 23)	^a^ *p* = 0.002; ^b^ *p* = 0.012

## 5 Discussion

The main observation of this study is that 60 days of BR caused a significant loss in force generating capacity and a transition to faster contractile properties of leg muscles, which were in part mitigated by AG. In contrast to our hypothesis, neither substantial oxidative changes of total muscle proteins nor any significant alteration in systemic oxidant/antioxidant status were found. Overall, muscle cell protection was not hampered during the 60 days of BR, while SOD activity was maintained with AG but increased at the end of BR in the absence of AG. Previously, for the same AGBRESA subjects, we showed a rapid development of fibre atrophy within the first 6 days of unloading, followed by a much slower rate between 6 and 55 days of BR [26]. Altogether, our data suggest that the loss in muscle force found following BR in the AGBRESA study was a consequence of muscle fibre atrophy [26], without a significant contribution of oxidative stress.

### 5.1 Effect of BR and AG on Muscle Functions

It was not surprising that 60 days of BR caused a significant reduction in maximal voluntary isometric KE force and electrically evoked twitch force of leg muscles. These results are in line with what has previously been found in other BR studies [2, 4, 10, S17] and by Kramer et al. [23], in the same subjects. In addition, we found that 60 days of BR significantly reduced the maximal isometric knee flexor strength, something that has received limited attention in the literature, likely because the knee flexors are neither postural nor anti‐gravitational muscles. However, some BR studies showed the development of substantial muscle atrophy in the knee flexors both following a few days of BR [S18] up to prolonged BR [27].

As the force generated during a contraction depends on the number of active interactions between actin and myosin per muscle cross‐sectional area [28, S19], the reduction in the peak torque was most likely the result of muscle fibre atrophy [26]. Overall, AG did not attenuate the reduction in maximal isometric strength of both muscle groups, as reflected by a similar % decline in the maximal strength in the AG group as compared to the Control group (Figure S2).

BR also affected the intrinsic contractile properties of leg muscles as indicated by the slow‐to‐fast transition in twitch contractile properties in PFs and KE muscles.

Previously, however, and in line with other BR studies [S20, S21], a reduction in voluntary RFD was reported for the same subjects [23] suggesting a slowing of the muscle. The RFD depicts the ability to quickly produce force or power, and it is thought to be influenced by the level of neural activation, fibre‐type composition and the mechanical properties of the tendon [S2]. Here, we evaluated the effect of BR on RFD during electrically evoked twitch contractions, which by bypassing the central neural control, allowed us to assess the contribution of muscle contractile properties that influence the voluntary RFD [S22]. While we found a lower RFD in absolute terms [23], the twitch RFD normalised to the peak force showed an increase, rather than a decrease in RFD, suggesting faster, rather than slower contractile properties, corresponding with the increase in maximal unloaded shortening velocity reported previously [S20].

The relaxation rate is determined by the rates of sarcoplasmic reticulum Ca^2+^ re‐uptake, Ca^2+^ dissociation from the myofibrillar proteins and cross‐bridge detachment [28, S19, S23]. Fast (type II) fibres have faster contraction and relaxation times than type I fibres [S22]. As we did not see a significant slow‐to‐fast transition in fibre type composition after BR in our previous study on these subjects [26], we suggest that an increased rate of sarcoplasmic reticulum Ca^2+^ re‐uptake through increased sarco (endo)plasmic reticulum Ca^2+^‐ATPase (SERCA) expression, which was previously described after a 23‐day unilateral lower limb suspension study [S24], may have underlined the faster contractile properties after BR in the current study. Previously Kramer et al. [23] showed that AG attenuated the effects of BR on plantar flexion strength, in the same subjects. Here, we provided further evidence that AG was effective in attenuating the reduction in the electrically evoked twitch peak torque in the PFs, but not in the KEs, while it attenuated the increased relaxation rate in the KEs but not in the PFs. The cause of this different effect of AG on muscle types is unclear, but it may be due in part to differing muscle pump activity of thigh and calf muscles observed during AG exposure in some, but not all subjects, to prevent presyncope symptoms [23, S9]. Subjective evidence suggests that subjects favoured calf muscle activity over KE activity, which might have contributed to the AG effect in attenuating the loss of twitch force in PFs but not in KEs. Without having a normalised dataset of muscle activity recording during AG [S9], this remains speculative, and it would also not fully explain the differences in response in contraction velocity between muscle groups.

Previously, we found that during either a series of isometric contraction or repetitive isotonic shortening contractions, the fatigue resistance of skeletal muscle was not affected following 21 days of BR [4, 29]. In the present study, we found that even after 60 days of BR, there was no significant change in muscle fatigue resistance during a sustained isometric contraction. Conversely, during a series of maximum isokinetic knee extension and knee flexion contractions, the fatigue resistance was significantly increased. It therefore appears that the isokinetic fatigue resistance is improved after a long period of BR.

Of note, the observed discrepancy between isometric and isokinetic fatigue resistance is analogous to the age‐related changes in fatigue resistance, where older people had a lower fatigue resistance during shortening contractions at high velocity, but a higher fatigue resistance during repeated or sustained isometric contractions [S25]. The authors suggested that the extent of muscle fatigue depends on the contraction mode and the force‐velocity properties of the muscle under study [S25]. This would match our observation where faster contractile properties after BR (opposite to the age‐related slowing) are associated with a higher fatigue resistance during shortening contractions.

Literature on sex differences in the response to BR in the intrinsic muscle contractile properties is scarce. Previously, Kramer et al. [23] observed following BR a more pronounced % decrease from baseline in jump power and maximal KE strength in women than in men. Generally, we did not find significant differences in the effects of BR on muscle strength or other muscle functional parameters between women and men. The only difference we observed was an elongated EMD following a twitch stimulus after BR in the KEs in women but not in men. EMD is a measure of the time lag between muscle activation and muscle force production and is inversely related to muscle‐tendon stiffness [S26‐S28], and an elongated delay may make normal daily life movement more difficult [S28]. The longer electrically evoked EMD in women following BR may indicate a disuse‐induced reduction in muscle‐tendon stiffness and may result in a slower reaction to perturbations in women following prolonged BR [S2, S28]. This would necessitate particular attention once daily motor‐activities are restarted after a period of muscle disuse [S2] or spaceflight, particularly in women.

### 5.2 Effect of BR and AG on Systemic and Muscle Oxidant/Antioxidant Status

Protein carbonylation is the most common protein modification that occurs as a result of oxidative stress. It takes place directly by oxidation of amino acids or indirectly by reaction with reactive carbonyl species derived from lipid oxidation [S29]. In line with previous BR studies [8, 10, S5, S6], we found that neither short‐ nor long‐term BR resulted in a significant change in the level of muscle carbonylated proteins or serum levels of oxidative stress. Despite the absence of significant alterations in the level of muscle protein carbonylation, we found a general significant relationship between the serum levels of 8‐iso‐PGF2α, an important marker of increased oxidative stress and lipid peroxidation, and isokinetic KE muscle fatigability. The cause of this relationship warrants further investigations.

In accordance with previous observations [8, 30, S5], we found that the activity level of serum SOD was unchanged through the BR period. As the activity of SOD increases in the presence of reactive oxygen species (ROS), its unchanged serum activity further corroborates that the systemic oxidative stress status was not altered significantly after 60 days of BR. Similar to unaltered systemic SOD activity, muscle SOD activity was unchanged in early BR. However, it was elevated after 60 days of BR in Controls, but not in the AG group. This suggests that BR alone induced some oxidative stress in the muscle that was adequately alleviated by SOD to prevent significant carbonylation of myofibrillar proteins, while centrifugation even attenuated this apparently mild disuse‐induced oxidative stress. This assumption was further reflected by increases in transcript levels of key proteins involved either in the regulation of the production of free radicals (p66^(SHCA)^) or cell protection (NRF‐2 and OGG1) [S30‐S32], after prolonged (day 55), but not short‐term BR (Day 6). Like SOD, the upregulation of NRF‐2 and OGG1 following prolonged but not short‐term BR might have reflected the activation of a muscle counteracting response to redox imbalance during disuse [S12]. Something similar was also observed in other long‐ or medium‐term BR studies [9, S14, S31] and suggests that a slowly progressing small oxidant load may result in a subsequent recruitment of the antioxidant system during prolonged disuse that was sufficient to prevent significant protein carbonylation in our subjects.

### 5.3 Muscle Atrophy Determinants

Activation of the ATP–ubiquitin–proteasome pathway could contribute to muscle atrophy and the loss of strength in BR or spaceflight. It is well recognised that oxidative stress can activate the expression of atrophy/proteolytic genes such as atrogin‐1 and MuRF‐1 [S33]. In our study, we did not find any changes in mRNA expression of atrogin‐1 and MuRF‐1, nor any impact of AG during the time‐course of BR, despite an increase in protein breakdown found at the end of BR (Figure S4). Although there are clear correlations between the onset of muscle atrophy and the increase in atrogin‐1 and MuRF‐1, their expression may be transient [S34], which would explain why in our or other BR studies [2, S34], there was no evidence of a marked increase in their expression at later time points. However, this does not completely explain why we did not find any increase in atrogin‐1 and MuRF‐1 transcript levels after 6 days of BR where the rate of atrophy is the highest [26].

Other mechanisms may have therefore contributed to the fibre atrophy in these subjects. One such mechanism could be the increased expression of muscle myostatin [31, 32]. Myostatin negatively regulates muscle growth, impairing the activation of satellite cells and myogenic factors, and/or reducing protein synthesis and increasing muscle catabolism [32]. Consistent with previous observation from a 5‐day BR study [31], we found a significant increase in muscle myostatin mRNA levels, as early as after 6 days of BR, which remained elevated between 6 and 55 days of BR. Of note, the myostatin trend observed in our subjects was in accordance with the trend of fibre atrophy [26], corroborating the contribution of this myokine to muscle atrophy in our subjects.

### 5.4 Oxidative Stress and the Irisin–BDNF Axis

Our previous findings from the AGBRESA study showed (despite muscle fibre atrophy) no evidence for deterioration of the neuromuscular interaction by 60 days of BR, as reflected by unaffected circulating CAF levels [25] and preserved motor unit number estimates [33]. Based on previous literature [34, 35], we hypothesised that there would be a positive relationship between circulating levels of irisin and protection of the neuromuscular interaction. To test this hypothesis, we assessed to what extent serum levels of irisin correlated with the systemic oxidant status and NMJ integrity, depicted by the circulating levels of SOD, BDNF and CAF, respectively [36]. We found a significant relationship between irisin and BDNF levels with 8‐iso‐PGF2α and serum SOD after long‐ but not after short‐term BR, supporting the suggestion that irisin and BDNF play a role in modulating systemic oxidative stress during prolonged disuse [17, 36, 37, 38, 39].

Other BR studies [12, 19] reported reductions in irisin circulating levels following BR. However, the PGC‐1α–irisin–BDNF axis was not significantly affected in our study. Irisin is expressed both by muscle and adipose tissue, and some authors suggested that adipose tissue could contribute to irisin circulating levels during BR [18]. A larger contribution of adipose tissue, despite fibre atrophy, may explain such differences observed in irisin circulating levels between our and other BR studies [12, 18]. This indeed deserves further investigation.

Finally, we found an inverse relationship between irisin and CAF. Agrin is a basal lamina protein important for the clustering of nicotinic acetylcholine receptors and other proteins at the NMJ [37]. The presence of CAF in human serum, which is generated by neurotrypsin‐mediated cleavage of agrin, represents a potential indication of NMJ fragmentation and has been found to be increased in sarcopenic subjects and muscle wasting conditions [ 36, 37]. Altogether, these data therefore let us speculate that high serum irisin levels together with BDNF may have had a protective role in maintaining neuromuscular integrity by minimising oxidative stress [17, 38, 39 ].

### 5.5 Study Limitation

As previously considered [23, S9], the main limitation of the present study was the relatively small sample size that may have resulted in a low statistical power for some of the analyses. Finally, fewer women than men were recruited, limiting the statistical power and the possibility of performing subgroup analyses to detect sex‐specific differences in the response to centrifugation. Furthermore, the muscle pump activity adopted by some subjects during the centrifugation runs [23, S9] might have masked the passive effect of AG on muscle function. Additionally, due to the low quantity of available muscle tissue, our muscle molecular analyses were limited to quantify only the mRNA levels and not the protein concentration or activity of key proteins involved in oxidant/antioxidant status, such as glutathione peroxidase, or the nitrosative stress level [S31], which could have given a broader picture of muscle oxidative status.

## 6 Conclusions

During BR, the loss in muscle function was not significantly related with alterations in oxidative stress. No evidence was found for increased protein carbonylation, or for significant alterations in local or systemic markers of oxidative stress. AG mitigated only some of the BR‐induced changes in muscle function, which leads us to suggest that AG exposure is not sufficient and hence needs to be combined with adequate exercise modalities to fully act as an effective countermeasure for microgravity‐induced decrements in muscular function [23, S35]. We have also provided some evidence for a relationship between the serum levels of the hormone irisin and the oxidant/antioxidant status, as well as the circulating levels of CAF, a biomarker of NMJ fragmentation. This is illustrated by the observation that the participants with the highest serum irisin and BDNF levels had indications of lower levels of systemic oxidative stress markers. More work needs to be conducted on the potential for irisin to be a biomarker of oxidative and metabolic status.

## Author Contributions

Study design: A.B., B.G., R.C.I.W., E.M. and H. D. Conceptualisation: A.B. Data collection: A.B., J.Z., M.V.C. and M.G. Muscle biopsy collection: B.G., and J.R. Data design of the ESA and NASA standard measures: E.M. Data analysis and interpretation: A.B. and H.D. Writing first draft: A.B. Writing, review and editing: A.B., B.G., E.M., M.G., M.V.C., J.Z., M.E., R.C.I.W., P. H. and H.D. Writing and editing final version: A. B and H.D. All Authors reviewed the manuscript and approved the final version. To the best of the authors' knowledge, author Jörn Rittweger fulfils the requirements stated in the policy and the ethical *guidelines* for *authorship* and publishing in JCSM.

## Ethics Statement

The AGBRESA study was approved by the Ethics Committee of the North Rhine Medical Association (reference number 2018143) in Düsseldorf, Germany. The AGBRESA study was registered in the German Clinical Trials Register (DRKS‐ID: DRKS00015677) and has therefore been performed in accordance with the ethical standards laid down in the 1964 Declaration of Helsinki and its later amendments. All participants gave written informed consent prior to their inclusion in the study. Details that might disclose the identity of the subjects under study have been omitted. The study complied with the ethical guidelines for authorship and publishing set by the *Journal of Cachexia, Sarcopenia and Muscle*.

## Conflicts of Interests

The authors declare no conflicts of interest.

## Supporting information


**Figure S1:** Study design. Before the start of 60 days of bed rest, participants spent 14 days in the bed rest facility (:envihab) for familiarisation and baseline data collection (BDC‐14 through BDC‐1). The participants were allocated to one of three groups: (1) cAG group, bed rest (BR) plus 30‐min continuous centrifugation (*n* = 8); (2) iAG group, BR plus 6 × 5 min centrifugation (*n* = 8); (3) a control (Controls) group, BR only, without AG (*n* = 8). After the 60 days of bed rest, participants were re‐ambulated and stayed for an additional 14 days in the bed rest facility for measurements and recovery (R + 0 through R + 13). Biopsies from vastus lateralis muscle and blood samples were taken at BDC‐1, HDT6 and HDT55 (muscle biopsies) or HDT57 (blood samples) (Days 6 and 55 or 57 head‐down tilt bed rest, respectively). Data on leg muscle performance were collected at BDC‐5 and BDC‐3 and R + 0 and R + 2. HDT, head‐down tilt bed rest. Figure created with BioRender.com.

**Figure S2:** Spaghetti plots of individual data points, and R + 2 normalised data on BDC maximal torque values recorded during repeated isometric knee extensions and flexions. (a) and (b) Spaghetti plots of individual data points and lines connecting subject baseline and R + 2 maximal torque values recorded during repeated isometric knee extensions (a) and flexions (b). (c) and (d) R + 2 normalised data on BDC maximal torque values recorded during repeated isometric knee extensions (c) and flexions (d). BDC: baseline data collected at 5 days before bed rest; R + 2, data collected at day 2 after the end of bed rest period; Controls, bed rest group without intervention; AG: cAG and iAG pooled subjects. Data are presented as mean ± SEM. Differences were considered significant at *p* < 0.05.

**Figure S3:** Spaghetti plots of %RFD and % RFR individual value between baseline and R + 0 recorded during electrically evoked muscle contraction of knee extensor and plantar flexor muscles. Twitch RFD (%RFD) and twitch RFR (%RFR) data were normalised for the maximal twitch force values recorded during electrical evoked twitch contraction. (a) %RFD and (b) % RFR recorded during electrically evoked muscle contraction of knee extensor muscles; (c) %RFD and (d) % RFR recorded during electrically evoked muscle contraction of plantar flexor muscles. BDC: baseline data collected at 3 days before the start of bed rest; R + 0, data collected after the end of the bed rest period; Controls, bed rest group without intervention; AG: cAG and iAG pooled subjects.

**Figure S4:** Urine 3‐methylhistidine. Irrespective of intervention with artificial gravity the urine 3‐methylhistine levels were significantly increased following bed rest, data indicating an increase in muscle protein breakdown. BDC: baseline data collected at 3 days before the start of bed rest; HDT60/R + 0, data collected after the end of the bed rest period; Controls, bed rest group without intervention; AG: cAG and iAG pooled subjects. Data are presented as means ± SEM. Differences were considered significant at *p* < 0.05.

**Figure S5:** Relationship between serum 8‐iso‐PGF2α and fatigue index of isokinetic knee extension. (a) pooled experimental time‐points (BDC‐1 + HDT6 + HDT57); (b) data collected at the end of the bed rest period (HDT57). BDC: baseline data collected at 1 day before the start of bed rest; HDT57, 57 days of bed rest. AG: cAG and iAG pooled subjects. Relationships were considered significant at *p* < 0.05.

**Figure S6:** Effect of BR and AG on irisin serum levels. Bed rest and centrifugation protocols did not significantly affect the serum levels of irisin (a). Controls, bed rest group without intervention; AG: cAG and iAG pooled subjects. (b) Spaghetti plot of serum irisin in each subject during the bed rest period. (c‐d) Relationship between serum irisin and brain‐derived neurotrophic factor (BDNF). (c) pooled experimental times points (BDC‐1 + HDT6 + HDT57); (d) data collected at the end of the bed rest period (HDT57). BDC‐1: baseline data collected 1 day before the start of bed rest; HDT6, 6 days of bed rest and HDT57, 57 days of bed rest. Data are presented as means ± SEM. Differences and relationships were considered significant at *p* < 0.05.

**Table S1:** Anthropometric data of the AGBRESA study participants at baseline (BDC).
**Table S2:** List of forward and reverse primers used for Real Time‐PCRs.
**Table S3:** Relationship between CAF and irisin serum levels before BR (Panel A) and after 6 days of BR (Panel B) Panel A, data collected at baseline (BDC‐1). Panel B, data collected at HDT6. AG, iAG + cAG pooled. Data are presented as mean ± SEM. Differences were considered significant at p < 0.05. a, third versus first tertile; b, third versus first tertile.

**Data S1:** Supplementary Reference List.
