# Supplementary figures and images for: Muscle Weakness and the Irisin–BDNF and Oxidative Stress Axis in the 60‐Day Pseudorandomised Controlled AGBRESA Bed Rest Study
Source: J Cachexia Sarcopenia Muscle. 2026 Mar 24;17(2):e70250. doi: 10.1002/jcsm.70250 (PMC13140337; doi:10.1002/jcsm.70250)

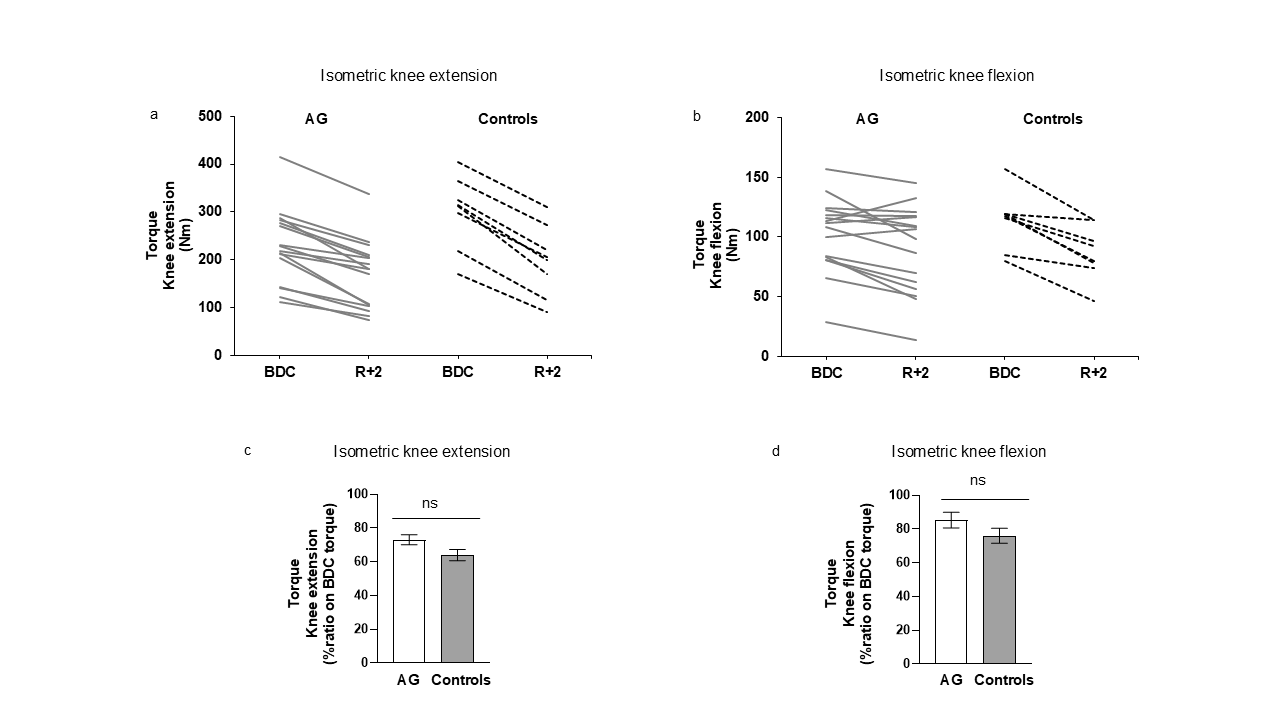

Supplement: Supplementary file 2 — Figure S2: Spaghetti plots of individual data points, and R + 2 normalised data on BDC maximal torque values recorded during repeated isometric knee extensions and flexions. (a) and (b) Spaghetti plots of individual data points and lines connecting subject baseline and R + 2 maximal torque values recorded during repeated isometric knee extensions (a) and flexions (b). (c) and (d) R + 2 normalised data on BDC maximal torque values recorded during repeated isometric knee extensions (c) and flexions (d). BDC: baseline data collected at 5 days before bed rest; R + 2, data collected at day 2 after the end of bed rest period; Controls, bed rest group without intervention; AG: cAG and iAG pooled subjects. Data are presented as mean ± SEM. Differences were considered significant at p < 0.05. [file JCSM-17-e70250-s008.tif]

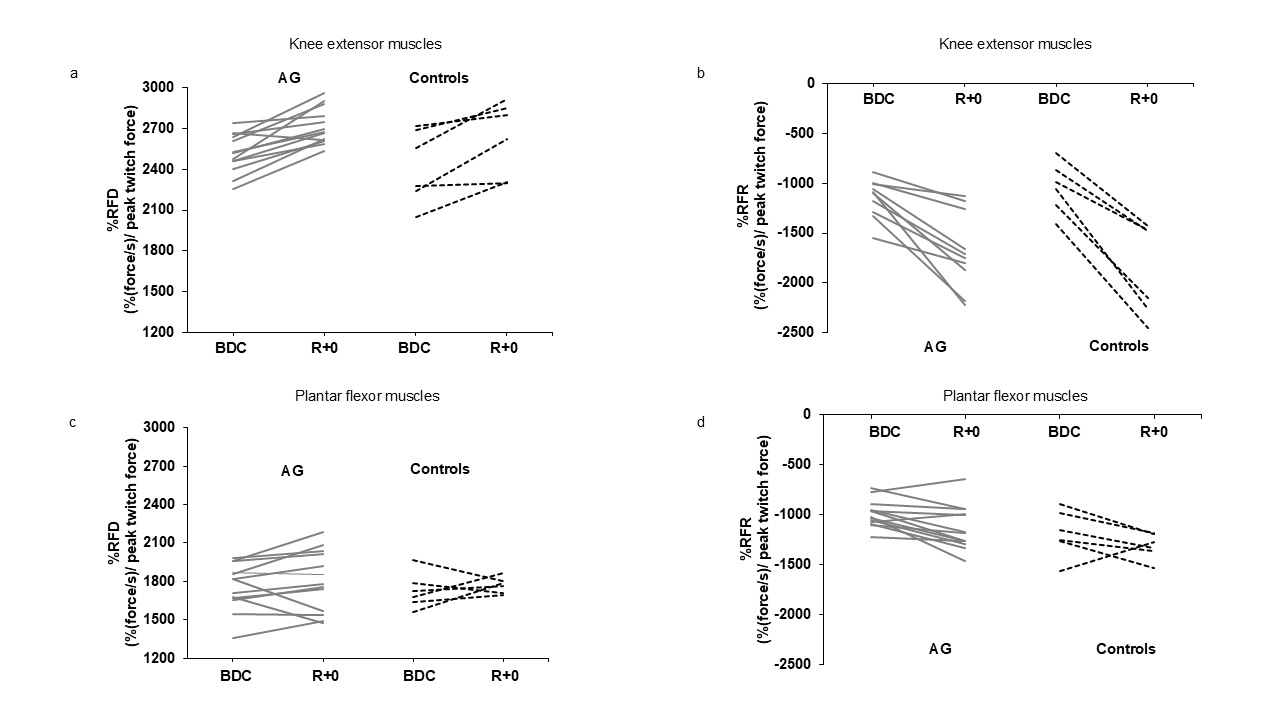

Supplement: Supplementary file 3 — Figure S3: Spaghetti plots of %RFD and % RFR individual value between baseline and R + 0 recorded during electrically evoked muscle contraction of knee extensor and plantar flexor muscles. Twitch RFD (%RFD) and twitch RFR (%RFR) data were normalised for the maximal twitch force values recorded during electrical evoked twitch contraction. (a) %RFD and (b) % RFR recorded during electrically evoked muscle contraction of knee extensor muscles; (c) %RFD and (d) % RFR recorded during electrically evoked muscle contraction of plantar flexor muscles. BDC: baseline data collected at 3 days before the start of bed rest; R + 0, data collected after the end of the bed rest period; Controls, bed rest group without intervention; AG: cAG and iAG pooled subjects. [file JCSM-17-e70250-s004.tif]

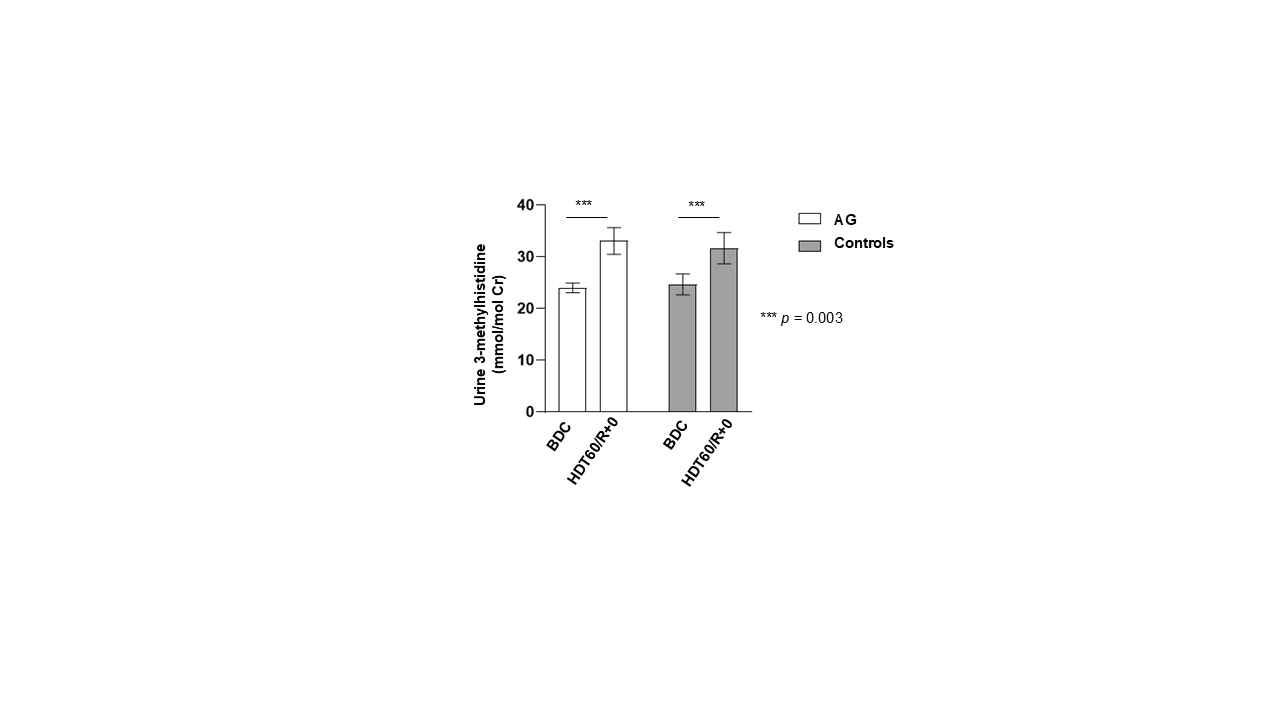

Supplement: Supplementary file 4 — Figure S4: Urine 3‐methylhistidine. Irrespective of intervention with artificial gravity the urine 3‐methylhistine levels were significantly increased following bed rest, data indicating an increase in muscle protein breakdown. BDC: baseline data collected at 3 days before the start of bed rest; HDT60/R + 0, data collected after the end of the bed rest period; Controls, bed rest group without intervention; AG: cAG and iAG pooled subjects. Data are presented as means ± SEM. Differences were considered significant at p < 0.05. [file JCSM-17-e70250-s005.tif]

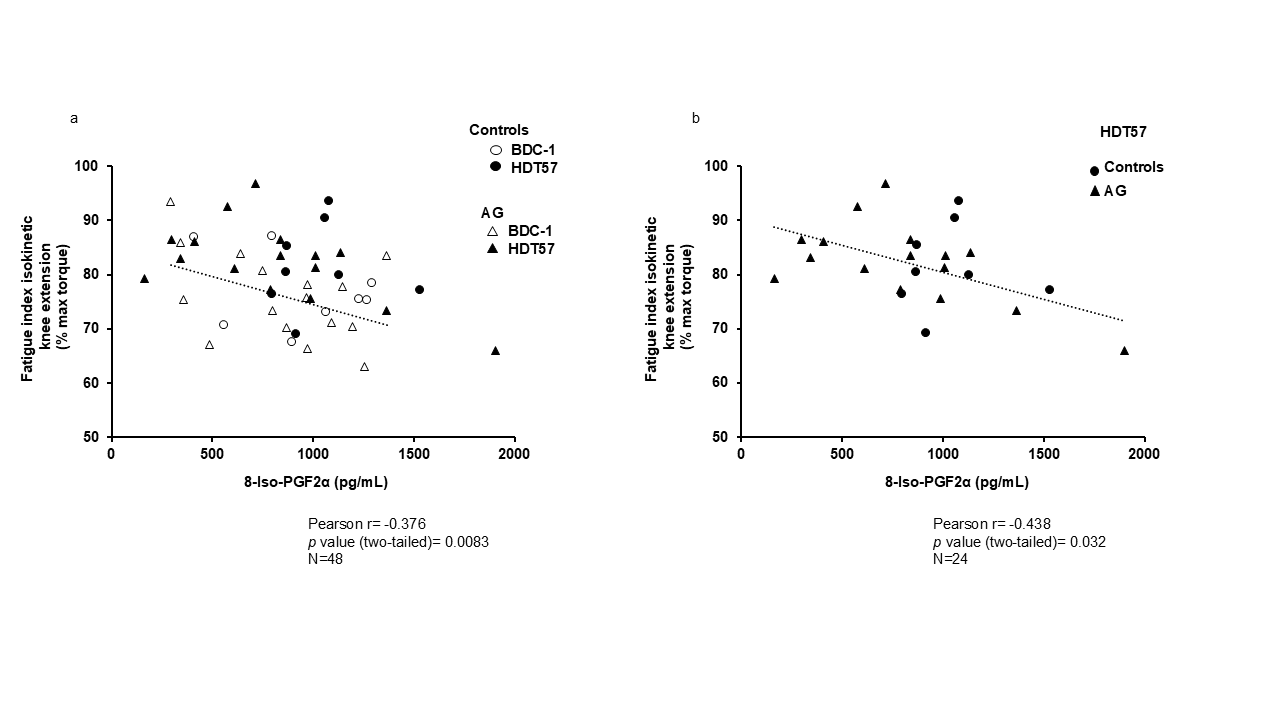

Supplement: Supplementary file 5 — Figure S5: Relationship between serum 8‐iso‐PGF2α and fatigue index of isokinetic knee extension. (a) pooled experimental time‐points (BDC‐1 + HDT6 + HDT57); (b) data collected at the end of the bed rest period (HDT57). BDC: baseline data collected at 1 day before the start of bed rest; HDT57, 57 days of bed rest. AG: cAG and iAG pooled subjects. Relationships were considered significant at p < 0.05. [file JCSM-17-e70250-s003.tif]
