# Supplementary material for: Muscle Weakness and the Irisin–BDNF and Oxidative Stress Axis in the 60‐Day Pseudorandomised Controlled AGBRESA Bed Rest Study
Source: J Cachexia Sarcopenia Muscle. 2026 Mar 24;17(2):e70250. doi: 10.1002/jcsm.70250 (PMC13140337; doi:10.1002/jcsm.70250)
Supplement: Supplementary file 7 — Table S1: Anthropometric data of the AGBRESA study participants at baseline (BDC). Table S2: List of forward and reverse primers used for Real Time‐PCRs. Table S3: Relationship between CAF and irisin serum levels before BR (Panel A) and after 6 days of BR (Panel B) Panel A, data collected at baseline (BDC‐1). Panel B, data collected at HDT6. AG, iAG + cAG pooled. Data are presented as mean ± SEM. Differences were considered significant at p < 0.05. a, third versus first tertile; b, third versus first tertile. [file JCSM-17-e70250-s001.docx]

**Journal of Cachexia, Sarcopenia and Muscle**

**Muscle weakness and the irisin-BDNF- and oxidative stress axis**

**in the 60-day pseudo-randomised controlled AGBRESA bed rest study**

Alessandra Bosutti^1*^, Bergita Ganse^2^, Edwin Mulder^3^, Markus Gruber^4^, Maria Venegas-Carro^5^, Jochen Zange^3^, Jörn Rittweger^3,†^, Moritz Eggelbusch^6,7^, Rob C.I. Wüst^6^, Paul Hendrickse^8^

and Hans Degens^9,10^

*^1^*Department of Life Sciences, University of Trieste, Trieste, Italy; *^2^*Saarland University, Innovative Implant Development (Fracture Healing), Departments and Institutes of Surgery, Homburg, Germany; *^3^*German Aerospace Centre (DLR), Institute of Aerospace Medicine, Cologne, Germany; *^4^*Human Performance Research Centre, Department of Sport Science, University Konstanz, Germany; *^5^*Applied Medical Informatics, Tübingen University Hospital, Germany; *^6^*Department of Human Movement Sciences, Faculty of Behavioural and Movement Sciences, Vrije Universiteit Amsterdam, The Netherlands; *^7^*Professorship of Exercise Biology, Department Health and Sport Sciences, TUM School of Medicine and Health, Technical University of Munich, Munich, Germany; *^8^*Lancaster Medical School, Lancaster University, Lancaster UK*;^9^*Department of Life Sciences, Manchester Metropolitan University, Manchester, UK; *^10^*Institute of Sport Science and Innovations, Lithuanian Sports University, Kaunas, Lithuania.

*Corresponding author:

Dr Alessandra Bosutti

Department of Life Sciences

University of Trieste

Via A. Valerio, n. 28/1

34127 Trieste,

Italy

Phone: +39(040)5588645

E-mail: alessandra.bosutti@units.it

ORCID: 0000-0002-8651-818X

**Supplementary Tables**

**Table S1 Anthropometric data of the AGBRESA study participants at baseline (BDC)**

|  | *N* | *Age (years)* | *Height (m)* | *Mass (kg)* | *BMI (kg/m^2^)* |
| --- | --- | --- | --- | --- | --- |
| *Controls* | 8 (6 m; 2 w) | 34.3±7.9 | 1.77±0.07 | 79.4±12.7 | 25.2±2.6 |
| *cAG* | 8 (5 m; 3 w) | 31.9±9.8 | 1.73±0.08 | 71.8±10.2 | 24.0±1.7 |
| *iAG* | 8 (5 m; 3 w) | 33.8±10.8 | 1.74±0.11 | 71.4±4.5 | 23.6±1.6 |
| *Pooled subject cohort* | 24 (16 m; 8 w) | 33.3±9.2 | 1.75±0.09 | 74.2±10.0 | 24.3±2.0 |

Controls: bed rest only; cAG: continuous artificial gravity (AG); iAG: intermittent AG. BMI: body mass index; m: men, w: women. Data are expressed as means ± SD. No significant differences were observed with respect to age, height or body mass [22].

**Table S2 List of forward and reverse primers used for Real Time‐PCRs**

| *Target* | *Forward primer* | *Reverse primer* |
| --- | --- | --- |
| *β-2-microglobulin*  *(NM_004048.4)* | AGA TGA GTA TGC CTG CCG TG | TTC AAA CCT CCA TGA TGC TGC |
| *GAPDH*  *(NM_002046.7)* | CACCATCTTCCAGGAGCGAG | CCTTCTCCATGGTGGTGAAGAC |
| *FNDC5*  *(NM_001171940.2)* | TCA TCG TCG TGG TCC TGT TC | TCA ATG ATG TCA TAC TGG CGG C |
| *PGC-1α*  *(NM_001330751.2)* | CACTTACAAGCCAAACCAACAAC | GGGAACCCTTGGGGTCATTT |
| *Myostatin*  *(NM_005259.3)* | CAG GCA CTG GTA TTT GGC AG | AAC GGA TTC AGC CCA TCT TCT C |
| *OGG1*  *(NM_002542.6)* | CAGACCAACAAGGAACTGGGA | CACTGAACAGCACCGCTTG |
| *P66Shc(A)*  *(NM_183001)* | CACGGGAGCTTTGTCAATAAGC | CCCCGGGTCCCATGACTTT |
| *Sirtuin-1*  *(AF083106.2)* | TAGAGCCTCACATGCAAGCTCTA | GCCAATCATAAGATGTTGCTGAAC |
| *NRF-2*  *(NM_006164.4)* | CACAGAAGACCCCAACCAGT | CTGTGCTTTCAGGGTGGTTT |
| *MuRF-1*  *(NM_032588.4)* | CCTGAGAGCCATTGACTTTGG | CTTCCCTTCTGTGGACTCTTCCT |
| *Atrogin-1*  *(NM_058229.4)* | GCAGCTGAACAACATT | CAGCCTCTGCATGATGTTCAGT |
| *Heme-Oxygenase*  *(NM_002133.2)* | GCCAGCAACAAAGTGCAAGAT | AGTGTAAGGACCCATCGGAGAA |

**Table S3 Relationship between CAF and irisin serum levels before BR (Panel A) and after 6 days of BR (Panel B)**

| *Serum irisin tertiles (µg/mL)* | | | | |
| --- | --- | --- | --- | --- |
| *Panel A*  *BDC-1*  *(Controls+AG)* | | | | |
|  | 1^st^ irisin tertile  (7.71-10.74 µg/mL) | 2^nd^ irisin tertile  (11.47-16.91 µg/mL) | 3^rd^ irisin tertile  (17.96-23.92 µg/mL) |  |
|  | mean±SEM | mean±SEM | mean±SEM |  |
| *CAF (pg/mL)* | 354±227  (*n*=8) | 324±166  (*n*=8) | 48±8^a,b^  (*n*=8) | ^a,^ *p* = 0.017;  ^b, c,^ *p* = 0.002 |
| *Panel B*  *HDT6*  *(Controls+AG)* | | | | |
|  | 1^st^ irisin tertile  (5.93-11.86 µg/mL) | 2^nd^ irisin tertile  (11.88-16.45 µg/mL) | 3^rd^ irisin tertile  (18.03-23.51 µg/mL) |  |
|  | mean±SEM | mean±SEM | mean±SEM |  |
| *CAF (pg/mL)* | 269±142  (*n*=8) | 353±190  (*n*=8) | 53±12^a,b^  (*n*=8) | ^a,^ *p* = 0.028;  ^b, c,^ *p* = 0.005 |
