# Supplementary material for: Muscle Weakness and the Irisin–BDNF and Oxidative Stress Axis in the 60‐Day Pseudorandomised Controlled AGBRESA Bed Rest Study
Source: J Cachexia Sarcopenia Muscle. 2026 Mar 24;17(2):e70250. doi: 10.1002/jcsm.70250 (PMC13140337; doi:10.1002/jcsm.70250)
Supplement: Supplementary file 8 — Data S1: Supplementary Reference List. [file JCSM-17-e70250-s006.docx]

**Muscle weakness and the irisin-BDNF- and oxidative stress axis**

**in the 60-day pseudo-randomised controlled AGBRESA bed rest study**

Alessandra Bosutti^1*^, Bergita Ganse^2^, Edwin Mulder^3^, Markus Gruber^4^, Maria Venegas-Carro^5^, Jochen Zange^3^, Jörn Rittweger^3,†^, Moritz Eggelbusch^6,7^, Rob C.I. Wüst^6^, Paul Hendrickse^8^

and Hans Degens^9,10^

*^1^*Department of Life Sciences, University of Trieste, Trieste, Italy; *^2^*Saarland University, Innovative Implant Development (Fracture Healing), Departments and Institutes of Surgery, Homburg, Germany; *^3^*German Aerospace Centre (DLR), Institute of Aerospace Medicine, Cologne, Germany; *^4^*Human Performance Research Centre, Department of Sport Science, University Konstanz, Germany; *^5^*Applied Medical Informatics, Tübingen University Hospital, Germany; *^6^*Department of Human Movement Sciences, Faculty of Behavioural and Movement Sciences, Vrije Universiteit Amsterdam, The Netherlands; *^7^*Professorship of Exercise Biology, Department Health and Sport Sciences, TUM School of Medicine and Health, Technical University of Munich, Munich, Germany; *^8^*Lancaster Medical School, Lancaster University, Lancaster UK*;^9^*Department of Life Sciences, Manchester Metropolitan University, Manchester, UK; *^10^*Institute of Sport Science and Innovations, Lithuanian Sports University, Kaunas, Lithuania.

*Corresponding author:

Dr Alessandra Bosutti

Department of Life Sciences

University of Trieste

Via A. Valerio, n. 28/1

34127 Trieste,

Italy

Phone: +39(040)5588645

E-mail: alessandra.bosutti@units.it

ORCID: 0000-0002-8651-818X

**Supplementary Reference List**

S1. Trappe S, Trappe T, Gallagher P, Harber M, Alkner B, Tesch P. Human single muscle fibre function with 84 day bed-rest and resistance exercise. J Physiol. 2004 Jun 1;557(Pt 2):501-13. doi: 10.1113/jphysiol.2004.062166. Epub 2004 Apr 2. PMID: 15064323

S2. Ruggiero L, Gruber M. Neuromuscular mechanisms for the fast decline in rate of force development with muscle disuse - a narrative review. J Physiol. 2024 Oct 28. doi: 10.1113/JP285667. Epub ahead of print. PMID: 39467095.

S3. Deschenes MR. Motor unit and neuromuscular junction remodeling with aging. Curr Aging Sci. 2011, 4:209- PMCID: PMC1665105.

S4. Maréchal G, Beckers-Bleukx G. Effect of nitric oxide on the maximal velocity of shortening of a mouse skeletal muscle. Pflugers Arch. 1998 Nov;436(6):906-13. doi: 10.1007/s004240050722. PMID: 9799406.

S5. Morgan JL, Zwart SR, Heer M, Ploutz-Snyder R, Ericson K, Smith SM. Bone metabolism and nutritional status during 30-day head-down-tilt bed rest. J Appl Physiol (1985). 2012 Nov;113(10):1519-29. doi: 10.1152/japplphysiol.01064.2012. Epub 2012 Sep 20. Erratum in: J Appl Physiol (1985). 2014 May 15;116(10):1343. PMID: 22995395; PMCID: PMC3524659.

S6. Dirks ML, Wall BT, van de Valk B, Holloway TM, Holloway GP, Chabowski A, Goossens GH, van Loon LJ. One Week of Bed Rest Leads to Substantial Muscle Atrophy and Induces Whole-Body Insulin Resistance in the Absence of Skeletal Muscle Lipid Accumulation. Diabetes. 2016 Oct;65(10):2862-75. doi: 10.2337/db15-1661. Epub 2016 Jun 29. PMID: 27358494.

S7. Park SE, Park CY, Sweeney G. Biomarkers of insulin sensitivity and insulin resistance: Past, present and future. Crit Rev Clin Lab Sci. 2015;52(4):180-90. doi: 10.3109/10408363.2015.1023429. Epub 2015 Jun 4. PMID: 26042993.

S8. Ren W, Xu Z, Pan S, Ma Y, Li H, Wu F, Bo W, Cai M, Tian Z. Irisin and ALCAT1 mediated aerobic exercise-alleviated oxidative stress and apoptosis in skeletal muscle of mice with myocardial infarction. Free Radic Biol Med. 2022 Nov 20;193(Pt 2):526-537. doi: 10.1016/j.freeradbiomed.2022.10.321. Epub 2022 Nov 3. PMID: 36336228.

S9. Kramer A, Venegas-Carro M, Mulder E, Lee JK, Moreno-Villanueva M, Bürkle A, Gruber M. Cardiorespiratory and Neuromuscular Demand of Daily Centrifugation: Results From the 60-Day AGBRESA Bed Rest Study. Front Physiol. 2020 Sep 11;11:562377. doi: 10.3389/fphys.2020.562377. PMID: 33041861; PMCID: PMC7518067.

S10 Place N, Casartelli N, Glatthorn JF, Maffiuletti NA. Comparison of quadriceps inactivation between nerve and muscle stimulation. Muscle Nerve. 2010 Dec;42(6):894-900. doi: 10.1002/mus.21776. Epub 2010 Oct 6. PMID: 20928903.

**S11.** Bourdier P, Zahariev A, Rudwill F, Kenny H, O’Gorman DJ, Chery I, Bareille MP, Koch GG, Sylvie Normand, Blanc S, Bergouignan A, Simon C. Effect of resistive exercise combined with vibration on body composition and energy balance – Results from the 21-day MNX bedrest study. Acta Astronautica, Volume 232,2025, Pages 387-396, ISSN 0094-5765. https://doi.org/10.1016/j.actaastro.2025.03.029

**S12.** Duchateau J. Bed rest induces neural and contractile adaptations in triceps surae. Med Sci Sports Exerc. 1995 Dec;27(12):1581-9. PMID: 8614311.

S1**3**. Blottner D, Capitanio D, Trautmann G, Furlan S, Gambara G, Moriggi M, Block K, Barbacini P, Torretta E, Py G, Chopard A, Vida I, Volpe P, Gelfi C, Salanova M. Nitrosative Redox Homeostasis and Antioxidant Response Defense in Disused *Vastus lateralis* Muscle in Long-Term Bedrest (Toulouse Cocktail Study). Antioxidants (Basel). 2021 Mar 3;10(3):378. doi: 10.3390/antiox10030378. PMID: 33802593; PMCID: PMC8001160.

S1**4**. Brocca L, Cannavino J, Coletto L, Biolo G, Sandri M, Bottinelli R, Pellegrino MA. The time course of the adaptations of human muscle proteome to bed rest and the underlying mechanisms. J Physiol. 2012 Oct 15;590(20):5211-30. doi: 10.1113/jphysiol.2012.240267. Epub 2012 Jul 30. PMID: 22848045; PMCID: PMC3497573.

**S15.** Persson M, Steinz MM, Westerblad H, Lanner JT, Rassier DE. Force generated by myosin cross-bridges is reduced in myofibrils exposed to ROS/RNS. Am J Physiol Cell Physiol. 2019 Dec 1;317(6):C1304-C1312. doi: 10.1152/ajpcell.00272.2019. Epub 2019 Sep 25. PMID: 31553646.

S1**6**. Singh CK, Chhabra G, Ndiaye MA, Garcia-Peterson LM, Mack NJ, Ahmad N. The Role of Sirtuins in Antioxidant and Redox Signaling. Antioxid Redox Signal. 2018 Mar 10;28(8):643-661. doi: 10.1089/ars.2017.7290. Epub 2017 Oct 20. PMID: 28891317; PMCID: PMC5824489.

S1**7**. Kawakami Y, Akima H, Kubo K, Muraoka Y, Hasegawa H, Kouzaki M, Imai M, Suzuki Y, Gunji A, Kanehisa H, Fukunaga T. Changes in muscle size, architecture, and neural activation after 20 days of bed rest with and without resistance exercise. Eur J Appl Physiol. 2001 Jan-Feb;84(1-2):7-12. doi: 10.1007/s004210000330. PMID: 11394257.

S1**8**. Franchi MV, Sarto F, Simunič B, Pišot R, Narici MV. Early Changes of Hamstrings Morphology and Contractile Properties during 10 d of Complete Inactivity. Med Sci Sports Exerc. 2022 Aug 1;54(8):1346-1354. doi: 10.1249/MSS.0000000000002922. Epub 2022 Mar 23. PMID: 35324511.

S1**9**. Koryak YA. Influence of simulated microgravity on mechanical properties in the human triceps surae muscle in vivo. I: effect of 120 days of bed-rest without physical training on human muscle musculo-tendinous stiffness and contractile properties in young women. Eur J Appl Physiol. 2014 May;114(5):1025-36. doi: 10.1007/s00421-014-2818-9. Epub 2014 Feb 8. PMID: 24509917; PMCID: PMC3983899.

**S20.** Adams GR, Caiozzo VJ, Baldwin KM. Skeletal muscle unweighting: spaceflight and ground-based models. J Appl Physiol (1985). 2003 Dec;95(6):2185-201. doi: 10.1152/japplphysiol.00346.2003. PMID: 14600160.

**S21.** Funato K, Matsuo A, Yata H, Akima H, Suzuki Y, Gunji A, Fukunaga T. Changes in force-velocity and power output of upper and lower extremity musculature in young subjects following 20 days bed rest. J Gravit Physiol. 1997 Jan;4(1):S22-30. PMID: 11541172.]

**S22.** Maffiuletti NA, Aagaard P, Blazevich AJ, Folland J, Tillin N, Duchateau J. Rate of force development: physiological and methodological considerations. Eur J Appl Physiol. 2016 Jun;116(6):1091-116. doi: 10.1007/s00421-016-3346-6. Epub 2016 Mar 3. PMID: 26941023; PMCID: PMC4875063.

S**23**. Wiles CM, Young A, Jones DA, Edwards RH. Relaxation rate of constituent muscle-fibre types in human quadriceps. Clin Sci (Lond). 1979 Jan;56(1):47-52. doi: 10.1042/cs0560047. PMID: 157828.

S**24**. Lamboley CR, Wyckelsma VL, Perry BD, McKenna MJ, Lamb GD. Effect of 23-day muscle disuse on sarcoplasmic reticulum Ca2+ properties and contractility in human type I and type II skeletal muscle fibers. J Appl Physiol (1985). 2016 Aug 1;121(2):483-92. doi: 10.1152/japplphysiol.00337.2016. Epub 2016 Jun 30. PMID: 27365282.

S**25.** Callahan DM, Kent-Braun JA. Effect of old age on human skeletal muscle force-velocity and fatigue properties. J Appl Physiol (1985). 2011 Nov;111(5):1345-52. doi: 10.1152/japplphysiol.00367.2011. Epub 2011 Aug 25. PMID: 21868683; PMCID: PMC3220307.

S2**6**. Hug F, Gallot T, Catheline S, Nordez A. Electromechanical delay in biceps brachii assessed by ultrafast ultrasonography. Muscle Nerve. 2011 Mar;43(3):441-3. doi: 10.1002/mus.21948. PMID: 21321958.

S2**7.** Cavanagh PR, Komi PV. Electromechanical delay in human skeletal muscle under concentric and eccentric contractions. Eur J Appl Physiol Occup Physiol. 1979 Nov;42(3):159-63. doi: 10.1007/BF00431022. PMID: 527577.

S2**8**. Blackburn JT, Bell DR, Norcross MF, Hudson JD, Engstrom LA. Comparison of hamstring neuromechanical properties between healthy males and females and the influence of musculotendinous stiffness. J Electromyogr Kinesiol. 2009 Oct;19(5):e362-9. doi: 10.1016/j.jelekin.2008.08.005. Epub 2008 Sep 30. PMID: 18829346.

S2**9**. Dalle-Donne I, Rossi R, Giustarini D, Milzani A, Colombo R. Protein carbonyl groups as biomarkers of oxidative stress. Clin Chim Acta. 2003 Mar;329(1-2):23-38. doi: 10.1016/s0009-8981(03)00003-2. PMID: 12589963.

S**30**. Giorgio M, Migliaccio E, Orsini F, Paolucci D, Moroni M, Contursi C, Pelliccia G, Luzi L, Minucci S, Marcaccio M, Pinton P, Rizzuto R, Bernardi P, Paolucci F, Pelicci PG. Electron transfer between cytochrome c and p66Shc generates reactive oxygen species that trigger mitochondrial apoptosis. Cell. 2005 Jul 29;122(2):221-33. doi: 10.1016/j.cell.2005.05.011. PMID: 16051147.

S**31**. Salanova M, Schiffl G, Gutsmann M, Felsenberg D, Furlan S, Volpe P, Clarke A, Blottner D. Nitrosative stress in human skeletal muscle attenuated by exercise countermeasure after chronic disuse. Redox Biol. 2013 Oct 28;1(1):514-26. doi: 10.1016/j.redox.2013.10.006. PMID: 24251120; PMCID: PMC3830069.

S**32**. Renaudin X, Campalans A. Modulation of OGG1 enzymatic activities by small molecules, promising tools and current challenges. DNA Repair (Amst). 2025 May;149:103827. doi: 10.1016/j.dnarep.2025.103827. Epub 2025 Mar 16. PMID: 40120404.

S**33**. Powers SK, Smuder AJ, Criswell DS. Mechanistic links between oxidative stress and disuse muscle atrophy. Antioxid Redox Signal. 2011 Nov 1;15(9):2519-28. doi: 10.1089/ars.2011.3973. Epub 2011 Jun 17. PMID: 21457104; PMCID: PMC3208252.

S**34**. Blottner D, Bosutti A, Degens H, Schiffl G, Gutsmann M, Buehlmeier J, Rittweger J, Ganse B, Heer M, Salanova M. Whey protein plus bicarbonate supplement has little effects on structural atrophy and proteolysis marker immunopatterns in skeletal muscle disuse during 21 days of bed rest. J Musculoskelet Neuronal Interact. 2014 Dec;14(4):432-44. PMID: 25524969.

S**35**. Hargens AR, Bhattacharya R, Schneider SM. Space physiology VI: exercise, artificial gravity, and countermeasure development for prolonged space flight. Eur J Appl Physiol. 2013 Sep;113(9):2183-92. doi: 10.1007/s00421-012-2523-5. Epub 2012 Oct 19. PMID: 23079865.
